# Genomic surveillance during the first two years of the COVID-19 pandemic – country experience and lessons learned from Türkiye

**DOI:** 10.3389/fpubh.2024.1332109

**Published:** 2024-05-24

**Authors:** Süleyman Yalçın, Yasemin Coşgun, Ege Dedeoğlu, Katharina Kopp, Fatma Bayrakdar, Gültekin Ünal, Biran Musul, Ekrem Sağtaş, Gülay Korukluoğlu, Philomena Raftery, Sedat Kaygusuz

**Affiliations:** ^1^National Molecular Microbiology Reference Laboratory, Public Health General Directorate, Ministry of Health, Ankara, Türkiye; ^2^Department of National Reference Laboratories and Biological Products, Public Health General Directorate, Ministry of Health, Ankara, Türkiye; ^3^National Virology Reference Laboratory, Public Health General Directorate, Ministry of Health, Ankara, Türkiye; ^4^World Health Organization Country Office, Ankara, Türkiye; ^5^Department of Medical Microbiology, University Health Sciences, Ankara Bilkent City Hospital, Ankara, Türkiye; ^6^Public Health General Directorate, Ministry of Health, Ankara, Türkiye

**Keywords:** severe acute respiratory syndrome coronavirus 2, COVID-19, genomic surveillance, next generation sequencing (NGS), public health

## Abstract

**Background:**

Türkiye confirmed its first case of SARS-CoV-2 on March 11, 2020, coinciding with the declaration of the global COVID-19 pandemic. Subsequently, Türkiye swiftly increased testing capacity and implemented genomic sequencing in 2020. This paper describes Türkiye’s journey of establishing genomic surveillance as a middle-income country with limited prior sequencing capacity and analyses sequencing data from the first two years of the pandemic. We highlight the achievements and challenges experienced and distill globally relevant lessons.

**Methods:**

We tracked the evolution of the COVID-19 pandemic in Türkiye from December 2020 to February 2022 through a timeline and analysed epidemiological, vaccination, and testing data. To investigate the phylodynamic and phylogeographic aspects of SARS-CoV-2, we used Nextstrain to analyze 31,629 high-quality genomes sampled from seven regions nationwide.

**Results:**

Türkiye’s epidemiological curve, mirroring global trends, featured four distinct waves, each coinciding with the emergence and spread of variants of concern (VOCs). Utilizing locally manufactured kits to expand testing capacity and introducing variant-specific quantitative reverse transcription polymerase chain reaction (RT-qPCR) tests developed in partnership with a private company was a strategic advantage in Türkiye, given the scarcity and fragmented global supply chain early in the pandemic. Türkiye contributed more than 86,000 genomic sequences to global databases by February 2022, ensuring that Turkish data was reflected globally. The synergy of variant-specific RT-qPCR kits and genomic sequencing enabled cost-effective monitoring of VOCs. However, data analysis was constrained by a weak sequencing sampling strategy and fragmented data management systems, limiting the application of sequencing data to guide the public health response. Phylodynamic analysis indicated that Türkiye’s geographical position as an international travel hub influenced both national and global transmission of each VOC despite travel restrictions.

**Conclusion:**

This paper provides valuable insights into the testing and genomic surveillance systems adopted by Türkiye during the COVID-19 pandemic, proposing important lessons for countries developing national systems. The findings underscore the need for robust testing and sampling strategies, streamlined sample referral, and integrated data management with metadata linkage and data quality crucial for impactful epidemiological analysis. We recommend developing national genomic surveillance strategies to guide sustainable and integrated expansion of capacities built for COVID-19 and to optimize the effective utilization of sequencing data for public health action.

## Introduction

Severe acute respiratory syndrome coronavirus 2 (SARS-CoV-2) was identified in Wuhan, Hubei, China, in late December of 2019 as the causative agent of an outbreak of pneumonia cases later termed Coronavirus disease 19 (COVID-19) by the World Health Organization (WHO) (1). Almost three months later, on March 11, 2020, WHO declared COVID-19 a global pandemic (2, 3). Türkiye holds a unique geographical position between Europe and Asia and is an international travel hub with a high population (> over 84.9 million people), and reported its first case of SARS-CoV-2 on the same day WHO declared the COVID-19 pandemic. The patient was reported as a Turkish male with recent travel in Europe (4). A few days later, on March 17, 2020, the first COVID-19-related death was reported, now known to have been a 90-year-old person who had contact with individuals from China (5).

In Türkiye, the Coronavirus Scientific Advisory Board (CSAB) was formed through a formal invitation by the Minister of Health Türkiye to its selected members (6). The CSAB functioned as an advisory committee within the Ministry of Health Türkiye (MoH-TR) to guide the national response strategy. They assisted in developing a series of guidance documents regarding case definitions and preventive measures for health workers (7, 8). Türkiye utilized its 2009 “National Pandemic Influenza Preparedness Plan,” which was updated in 2019, to guide the response (9). Throughout the two years of the pandemic, public health and social measures (PHSM) were implemented nationwide, including social isolation recommendations, mandatory mask-wearing, closure of schools, and isolation of high-risk individuals (10). These decisions were guided by the data generated through Hayat Eve Sığar (HES), a smartphone app developed by MoH-TR to increase surveillance capacity (11). The vaccination campaign in Türkiye commenced in January 2021, initially with the roll-out of the CoronaVac vaccine (Sinovac Life Sciences, Beijing, China) (12), with the first doses being administered on January 13, 2021. The MoH-TR developed a national vaccine administration strategy detailing priority groups in the society for vaccine administration (13). On March 24, 2021, 1.4 million doses of the BNT162b2 vaccine (Pfizer & BioNTech, USA & Germany) (14) were received. As of May 2022, approximately 70% of the Turkish population had been vaccinated with a single vaccine (CoronaVac, BNT162b2 & TurkoVac (15)), and approximately 60% were fully vaccinated.

Regional public health laboratories (seven in March 2020) with pre-existing molecular diagnostics testing capacity using polymerase chain reaction (qPCR) functioned as Biosafety level 2+ (BSL2+) facilities (16). In January 2020, Türkiye’s National Virology Reference Laboratory (NVRL) developed a quantitative reverse transcription PCR (RT-qPCR) kit in collaboration with a private diagnostic manufacturing company (Bioeksen R&D Technologies, Istanbul, Türkiye) at the a cost of $1.45 per test, with results available within 40 min (17). The diagnostic kit received WHO Emergency Use Authorization Listing (EUAL) on November 30, 2020 (18). By leveraging this pre-existing infrastructure, alongside an inexpensive and rapid RT-qPCR kit, Türkiye rapidly established testing capacity throughout the country.

Although next-generation sequencing (NGS) for SARS-CoV-2 was established at the NVRL in March 2020, due to limited staff capacity at the laboratory, NGS was not routinely conducted in the first year of the pandemic. Until then, 217 sequences were uploaded to GISAID (a global initiative on sharing all influenza data) (19) from Türkiye. The majority were uploaded by institutes or university hospitals with NGS capacity, and only a fraction were uploaded by the NVRL (n = 27). However, on December 18, 2020, WHO designated the Alpha variant as a variant of concern (VOC) (20), which prompted the MoH-TR to expand the use of NGS as part of the pandemic response. In addition, the global circulation of the Alpha VOC drove the rapid development of Türkiye’s first variant-specific detection kit. The Alpha VOC kit was developed to detect the N501Y mutation on the spike gene of SARS-CoV-2, which, at the time, was believed to be specific for Alpha VOC (21). To complement the development of the Alpha VOC kit on December 20, 2020, the NVRL (as the only government-authorized center for SARS-CoV-2 NGS in Türkiye) expanded NGS from December 24, 2020, to track SARS-CoV-2 mutations and circulation of variants in Türkiye.

As more VOCs were designated, nationally developed RT-qPCR kits were distributed to the regional laboratories in the surveillance network. The variant RT-qPCR kits could simultaneously detect characteristic mutations of the WHO-designated VOCs through multiplex RT-qPCR reactions. With limited knowledge of NGS in the public health sector then, this ability led Türkiye to cost-effectively detect and monitor the circulating VOCs in the country, utilizing the existing workforce in molecular diagnostics without the need for extensive sequencing capacity. From December 28, 2020 (when Türkiye started recording geographical information) to February 17, 2022, Türkiye had submitted 86,429 sequences to GISAID. Of these, the NVRL uploaded 86,137 (99.66%), with the remaining 292 sequences uploaded by university hospitals in Türkiye.

This study describes Türkiye’s experience establishing and implementing a genomic surveillance system as a middle-income country with limited prior knowledge of NGS. It presents an analysis of genomic surveillance data from the first two years of the pandemic. We highlight the achievements and challenges experienced and draw out globally relevant lessons.

## Methods

### Sample referral and testing strategy

**Figure 1:**
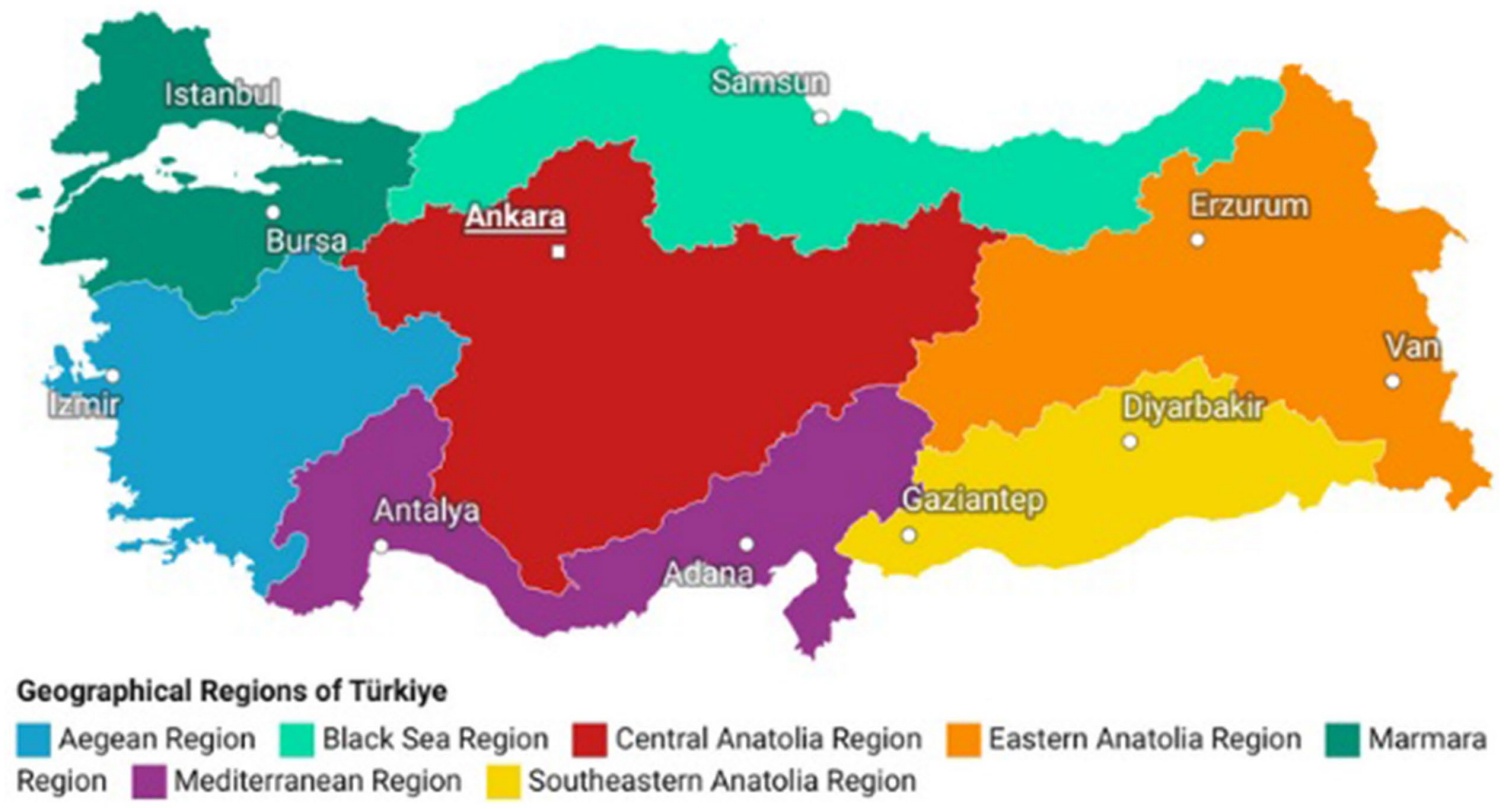
Map of Türkiye showing the country’s geographical regions and the locations of regional laboratories that refered samples to the NVRL for NGS. The laboratories included in the genomic surveillance network were from the Marmara region (dark green) (six laboratories from Istanbul and one laboratory from Bursa), the Aegean region (light blue) (one laboratory in Izmir), the Mediterranean region (purple) (two laboratories, one each in Antalya and Adana), Southeastern Anatolian region (yellow) (two laboratories, one each in Gaziantep and Diyarbakir), Eastern Anatolia region (orange) (two laboratories, one each in Van and Erzurum), Black Sea region (light green)(one laboratory in Samsun), and Central Anatolia region (red) (one laboratory in Ankara).

The samples referred to in this study were routine samples forwarded to the NVRL from the regional public health laboratories in Türkiye as part of the national genomic surveillance network. Initially, seven laboratories referred samples to the NVRL for NGS, which increased to 16 in 2021 to improve geographic representation (see Figure 1). The NVRL also received samples from government hospitals in areas where case numbers were high. Samples were collected using a nationally developed extraction buffer to extract and preserve nucleic acids from swabs while simultaneously inactivating the virus. Using these reagents was economical and increased SARS-CoV-2 testing efficiency by reducing the need for a separate extraction step without compromising sensitivity (22).

**Figure 2:**
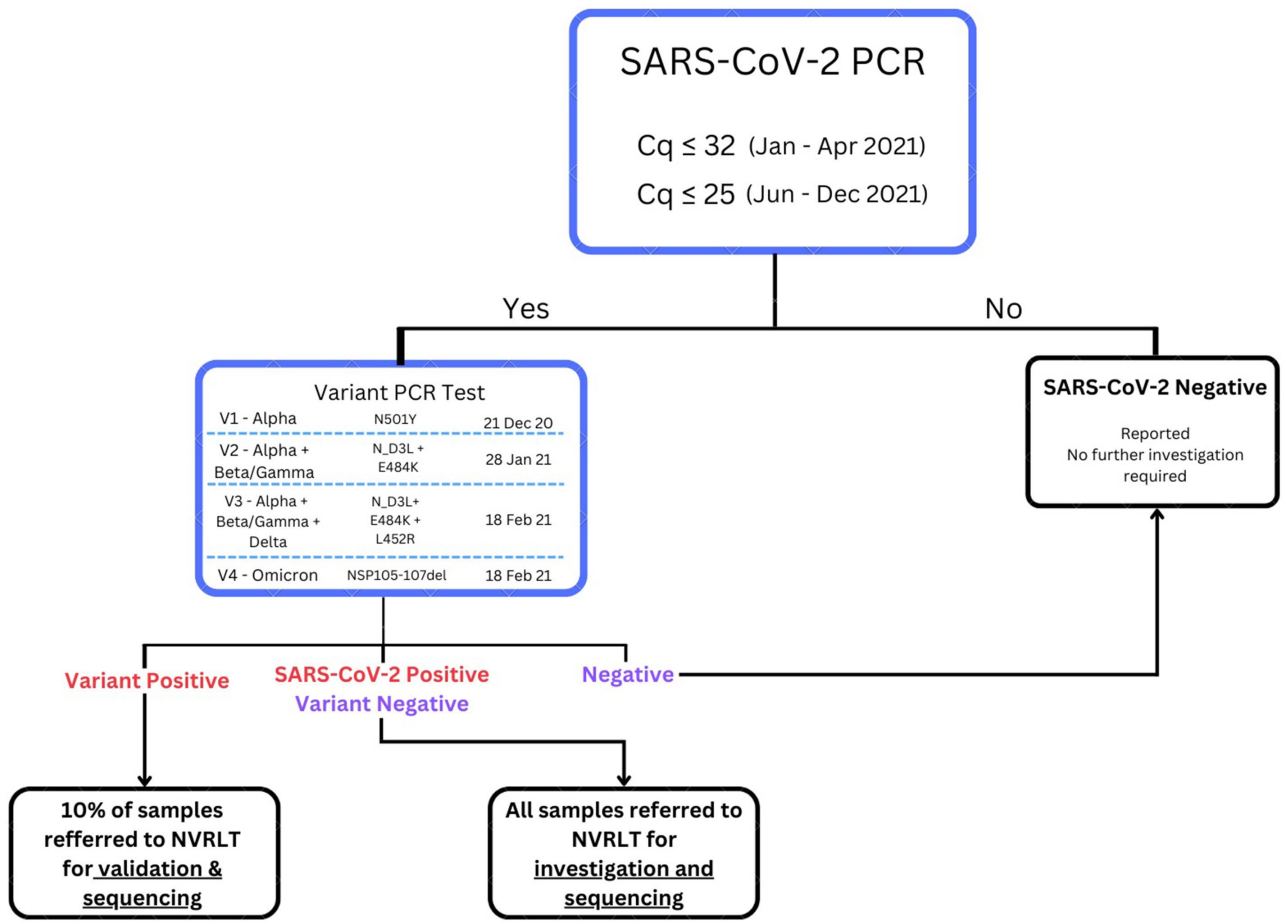
Flowchart of the sample referral system for SARS-CoV-2 sequencing in Türkiye. The sample referral system of Türkiye, where regional laboratories with variant RT-qPCR detection capacity referred samples for NGS to the NVRL. At the regional level, laboratories tested each sample, and positive samples were re-tested using variant RT-qPCR. Following confirmatory testing, if previously positive samples tested negative, this sample was reported as SARS-CoV-2 negative. All samples that tested negative for any of the mutations or tested positive for multiple mutations using the variant kits were referred to the NVRL for NGS. In addition, 10% of samples were referred for NGS to the NVRL if they tested positive for a single mutation.

Throughout the pandemic, the MOH-TR, in partnership with a private company, developed and updated four versions of the RT-qPCR kits for variant detection in response to WHO’s VOC designations. Briefly, these kits targeted (i) an internal control gene (*RnaseP*), (ii) a universal SARS-CoV-2 target, and (iii) characteristic mutation(s) of VOCs in a multiplex RT-qPCR reaction (see Figure 2). The first RT-qPCR kit detected the N501Y mutation and was designed to detect the Alpha variant. The second version detected N_D3L and E484K mutations, targeting Alpha and Beta/Gamma variants. The third version detected the N_D3L, E484K, and L452R mutations targeting Alpha, Beta/Gamma, and Delta VOCs, respectively. The fourth and final version of the kit targeted the Nsp106-107del mutation and focused only on the Omicron variant.

The referral algorithm for samples referred to the NVRL evolved over time. A flowchart summarizing the dates when the variant detection RT-qPCR kits were progressively utilized and the referral system to the NVRL is presented in Figure 2. All samples that tested negative for any of the mutations or tested positive for multiple mutations using the variant kits were referred to the NVRL for NGS. Additionally, 10% of positive cases were referred for NGS.

In Türkiye, a nasopharyngeal swab was collected from suspected COVID-19 cases and assigned a unique barcode, which was also used to report patients’ RT-qPCR results. Regional labs referred aliquots of samples to the NVRL for NGS. Which meant that samples were disconnected from their original barcode, and sequences could not be linked back to the patient details on the original samples. Once the sample arrived at the NVRL, the staff manually assigned each sample a six-digit code (referred to as NVRL-code). Therefore, samples referred to the NVRL were not linked with metadata (e.g., gender, age, vaccination status, disease state) apart from the referral laboratory and province. The NVRL manually kept an in-house (cloud-based MS Excel worksheet) sample information system (SIS) where the only information that was recorded was the (i) NVRL code, (ii) originating city, (iii) assigned lineage, and (iv) identified mutations. The NVRL staff successfully uploaded the fasta files of each sample and assigned a lineage to GISAID using the NVRL code as the primary identifier.

### NGS laboratory procedures

It was not feasible to perform direct sequencing from the extraction buffer used nationally, so any samples received in this buffer from regional labs had to undergo nucleic acid extraction prior to sequencing. To carry out this process, the NVRL employed three different automatic nucleic acid extractors. The first was the RINA M14, a robot that could extract total nucleic acid from 14 samples per hour. The second instrument, the Zybio 3,000, and its corresponding extraction kit (manufactured by Zybio Inc., China) yielded superior sequencing results and could process 32 samples in just 9 min. The primary nucleic acid extraction system used by the NVRL was the Zybio system due to its increased output and quality. However, for some samples requiring further investigation, the EZ1 Automatic extractor with EZ1 Viral Mini kits (manufactured by Qiagen, Germany) was used. The samples were sequenced in batches of 96 and were kept at +4°C during the sequencing process. Following sequencing, the extracted samples were stored at −80°C indefinitely.

All NGS for SARS-CoV-2 between December 21, 2020, and February 26, 2022, was conducted at the NVRL. NGS was performed using two Illumina platforms (MiSeq and NextSeq 550) and the Oxford Nanopore Technologies (ONT) GridION.

The NVRL used several kits for library preparation, including the following: (i) the CleanPlex SARS-CoV-2 kit (Paragon Genomics, United States), (ii) the Illumina COVIDSeq Test (Illumina, USA), and (iii) the Rapid Barcoding Kit (Oxford Nanopore Technologies, UK) with the Midnight primer scheme (21). Libraries were generated according to the manufacturer’s instructions for the CleanPlex and COVIDSeq kits. However, to lower sequencing costs, a private company (Bioeksen R&D Technologies, Türkiye) developed an experimental “cDNA and amplicon generation kit” named “47WGS” for the ONT system. The kit utilized 47 unique barcodes (termed indexes) that pooled 47 samples into a single tube, which could then be barcoded using a single ONT rapid barcode. According to the manufacturer’s instructions, the R9.4.1 flowcell could be used simultaneously for 470 samples (10 rapid barcodes) with 72 h of runtime. Although this increased the time required for sample reporting, it significantly reduced the cost per genome sequenced. The resulting raw reads of a 470-sample run were demultiplexed and further processed for consensus genome generation.

### Consensus genome generation, lineage, and VOC assignment

For the Illumina kits, the NVRL utilized manufacturer-recommended software solutions for (i) processing raw reads, (ii) assembling/mapping raw reads to the reference genome of SARS-CoV-2, (iii) consensus genome generation, (iv) variant calling, and v) lineage determination. For the CleanPlex kits, the Sophia Genetics (Lausanne, Switzerland) developed software solutions were utilized, while the DRAGEN COVID Lineage app was used for the COVIDSeq kits.

For the ONT runs, an alternate program was provided with the kits titled “ncov-analyzer” (Massive Bioinformatics R&D Technologies Inc., Izmir, Türkiye). The program was designed for the extra multiplexing steps of the 47WGS kit. It demultiplexed a single fastq file to up to 47 individual fastq files depending on an index sequence. After the generation of 470 fastq files, these fastq files were processed using the ARTIC networks published pipeline (23). Briefly, after reads were quality controlled and quality trimmed using FastQC (24) and Trimmomatic (25) (using Phred score ≥ 9), reads were mapped minimap2 (26) to the reference genome of SARS-CoV-2, MN908947.3 was conducted. After mapping, variant calling was performed with medaka (27), and consensus genome creation was conducted with an in-house algorithm.

### Retrieval, curation, and selection of genomic data and metadata

Türkiye’s genomic sequence data from December 28, 2020, to February 17, 2022, along with corresponding metadata, were downloaded from the GISAID platform (17). This resulted in an extensive data set (*n* = 86,429), referred to as the “86 k dataset” in the results section of this paper. The metadata information extracted from all entries for this study included the sample name, the EPI ID assigned by GISAID, the dates of sample collection and submission of the consensus genome sequence to GISAID, the viral lineage assigned by Pangolin, the clade as classified by the GISAID, and the sequencing technology used.

In the 86 k dataset, only 43,494 (50.3%) samples were linked to geographical information (province and region). The respective data set is referred to as the “43 k” dataset below for further analysis. For these samples collected from December 28, 2020, to February 17, 2022, the administrative province of the sampling site and its geographical region (Aegean, Black Sea, Central Anatolia, Eastern Anatolia, Marmara, Mediterranean, and Southeastern Anatolia) was recorded by the NVRL.

The 43 k dataset was filtered to obtain only high-quality genome sequences, ensuring high phylogenetic accuracy. The following filters were applied to the dataset: Genome sequences with less than 1% ambiguities and at least 99% of the length of the GISAID reference genome (hCoV-19/Wuhan/WIV04/2019|EPI_ISL_402124, length: 29891 b, no ambiguities). In addition, all sequences that were flagged with warnings after their submission to the GISAID EPICoVServer were excluded. The remaining 31,629 (“31 k dataset”) high-quality genome sequences were aligned to the WIV04 reference genome as aligned in the unmasked multiple sequence alignment (MSA) in GISAID. For this, mafft (28) (version 7.505–12,022/Apr/10, with parameters --inputorder --keeplength --compactmapout --maxambiguous 1.0 --addtotop <ownsequences> − -auto input) was used. Overhangs at the 5′- and 3′-end of the resulting MSA were trimmed so that all sequences started at position 93 and ended at position 29,790 of the GISAID reference genome (length: 29698 bp).

Before uploading to the GISAID repository, the viral lineage according to the Pangolin classification scheme (29, 30) and the Nextclade clade was determined for each consensus genome sequence using the web server version of Nextclade (31). The 43 k data sample and its subset 31 k are subsets of the 86 k. In addition, membership of one of the five currently known VOC classes (VOC: Alpha, Beta, Delta, Gamma, and Omicron) or variant of interest categories (VOI: Epsilon, Eta, Kappa. Mu) according to the WHO nomenclature based on Pangolin lineage was determined by an in-house parsing program.

Sequences of Pangolin lineages not associated with any of the WHO nomenclature VOC or VOI categories were classified as “NonVOCassigned” or “Others” in the following analysis. For further phylodynamic and phylogeographic analyses, the 31 k data set was further broken down by grouping samples into subsets of the seven regions of Türkiye and by their membership of one of the WHO nomenclature VOC or VOI categories.

### Phylogenetic, phylodynamic, and phylogeographic analyses

To investigate the phylodynamic and phylogeographic aspects of SARS-CoV-2 genome sequences from within Türkiye and globally, we used Nextstrain (32) to generate a country-specific build. Five thousand sequences of the 31 k data set were randomly selected by Nextstrain’s probabilistic “subsampling” approach and analyzed against ~1,000 randomly selected sequences from the European region (as assigned by GISAID metadata), excluding samples from Türkiye. This regional context random selection was based on a grouping by country, year, and month and Nextstrain’s definition of “proximity” to the 5,000 custom samples. Analogously, another global background of ~1,000 global sequences from countries outside the WHO European region was selected. The Nextstrain pipeline performs all analyses in one workflow, from submitting pathogen genome sequences and respective metadata to displaying the results of the phylogenetic analysis in a web browser visualization.

To generate the Nextstrain build for this study, submitted sequences and metadata were filtered using Nextstrain’s augur version 20.0.0 filter (32) and aligned by using its default settings of the multiple sequence alignment program mafft (28) preliminary and refined tree rooted in the reference genome (Wuhan Hu-1) was inferred by using IQTREE (33). Afterward, a molecular clock and an ancestral-state reconstruction (34) were performed. Finally, the results were visualized on a web browser using the auspice program of the Nextstrain tool suite. To investigate the genomic epidemiology of different viral variants, we chose to stratify our sample set by the membership of the WHO nomenclature VOC or VOI categories. Sequences that could not be assigned to a Pangolin lineage belonging to WHO VOC or VOI classes were grouped under “NonVOCassigned” or “Others” in the visualization of these results.

### Statistical tests

The Shapiro–Wilk test of normality (35) was used to infer the normality of the distribution of monthly case numbers and corresponding sequencing numbers. The Spearman rank correlation was done on the inverse normal distributed data (the monthly case numbers and sequencing numbers). All statistical tests were conducted using the R statistical software (36).

### Ethics

This study was conducted as part of the public health response to the COVID-19 pandemic, led by the MoH-TR, and therefore did not require ethical approval. All SARS-CoV-2 sequences and information (e.g., collection date, location) included in this analysis were obtained during the first-line testing, analysis, and sequencing of samples from suspected cases through the national response. All patient samples were de-identified, and no other individual-specific information was used in this study.

## Results

**Figure 3:**
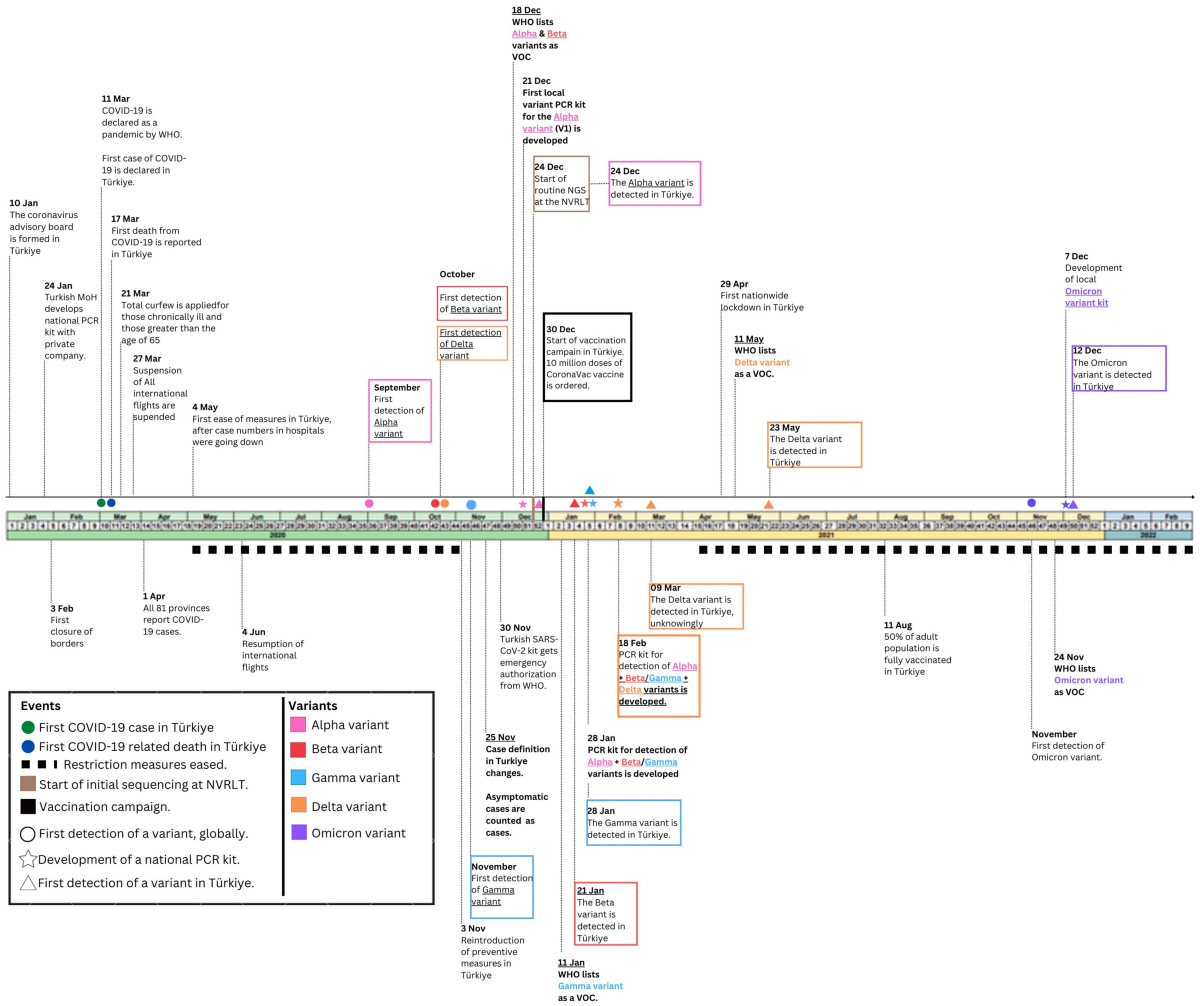
Timeline of COVID-19 pandemic related milestones in Türkiye. The timeline highlights the start of preventive measures and key milestones during the pandemic and includes time points of detecting VOCs globally and in Türkiye.

**Figure 4:**
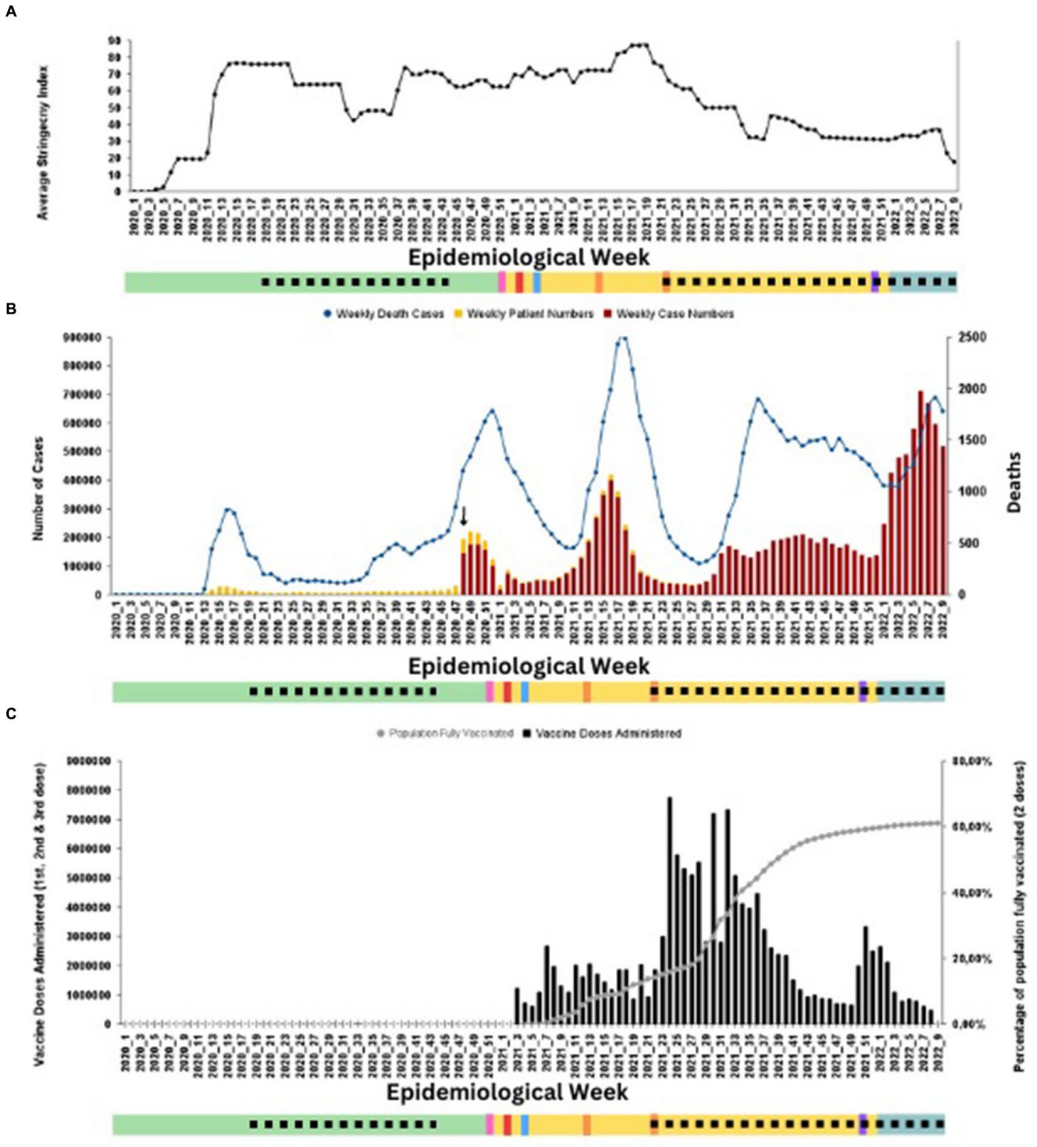
Türkiye’s **(A)** OxCGRT government PHSM stringency index against COVID-19, **(B)** Epidemiological curve, and **(C)** vaccination numbers, aggregated weekly, from January 2020–February 26, 2022. The timetable of events introduced in Figure 4 is present as the x-axis. Detection of VOCs are marked by their respective colours (Alpha-pink, Beta-red, Gamma-blue, Delta-orange, and Omicron-purple. **(A)** Displays the OxCGRT government PHSM stringency index against COVID-19. **(B)** Presents the epidemiological curve for Türkiye, showing the progression of reported weekly SARS-CoV-2 cases on the left y-axis (bar graphs, gold – symptomatic patients, red – positive case numbers) and deaths on the right y-axis (line graph, dark blue) in the same period. The black arrow indicates week 48, where the MoH-TR changed its reporting to include all RT-qPCR-positive cases irrespective of symptoms. **(C)** Displays Türkiye’s Vaccination data. Total vaccine administrations in population over 18 years of age - black bar graphs and percentage of the population completely vaccinated with two doses (grey line graph). All data for B and C were obtained from official MoH-TR sources and are publicly available.

In this section, we describe the evolution of the COVID-19 pandemic in Türkiye from January 2020 to February 2022 and present the results of the analysis of sequencing data produced during this period. Figure 3 presents a timeline of key milestones during the first two years of the COVID-19 pandemic in Türkiye (January 2020 to February 2022), and Figure 4 displays Türkiye’s COVID-19 PHSM stringency index, epidemiological curve, and vaccination numbers, aggregated weekly, from January 2020 to February 26, 2022.

### Evolution of the COVID-19 pandemic in Türkiye from January 2020 to February 2022

Selected parameters of the Turkish government’s response to mitigate the COVID-19 pandemic were extracted from the Oxford Covid-19 Government Response Tracker (OxCGRT) stringency index (24) (Figure 4A). With initial PHSMs implemented in the early days of the pandemic, case numbers and deaths in Türkiye remained lower than in the first weeks of the pandemic (Figure 4B). Reported case numbers and deaths had decreased by the end of April 2020. To limit the economic loss that the pandemic caused on tourism, based on advice from the CSAB, Türkiye eased measures on May 4, 2020. PHSMs remained relatively relaxed until November 3, 2020, when an increase in both cases and deaths occurred (Figure 4B).

Similar to global trends, the epidemiological curve in Türkiye showed four main waves during which case numbers and deaths increased, coinciding with the introduction and transmission of each novel VOC in the country (Figure 4B). Until November 25, 2020, official cases reported by the MOH included only symptomatic COVID-19 cases (Figures 3, 4B, black arrow). This explains the lack of an initial distinct wave in Türkiye’s epidemiological curve, as the case numbers until week 48 show a lower number of cases. The Alpha wave peaked with 220,667 cases and 1,781 deaths in week 48 and week 52, respectively (Figure 4B). As the emergence of the Alpha VOC prompted the MoH-TR to integrate sequencing in its surveillance strategy (Figure 3), it is difficult to discern from the available data the exact date of introduction of the Alpha VOC to Türkiye.

Implementation of PHSMs from week 43 of 2020 to week 19 of 2021 resulted in a decrease in case numbers. As was seen globally, the spread of VOCs Beta, Gamma, and Delta in Türkiye resulted in rises in case numbers and deaths during respective waves (Figure 4). Türkiye gradually relaxed PHSMs from week 22, 2021, as the numbers of vaccinated individuals increased despite this coinciding with the introduction and spread of the Delta VOC (Figure 4). Around the same time, the MoH began administering the BNT162b1 vaccine (Figures 3, 4). By the time the Delta VOC was circulating in Turkiye, a high vaccine coverage had been reached, reducing the death rate. The fourth and final peak coincided with the introduction and detection of the Omicron VOC in the country, with case numbers reaching a weekly high of 711,880 (Figures 3, 4).

### SARS-CoV-2 testing strategy

The detection of SARS-CoV-2 VOCs in Türkiye was facilitated by the rapid development of national RT-qPCR kits through close collaboration between the MoH-TR and a private company. Kits were shipped to the regional laboratories approximately 30 days (18.5 ± 16 days) after WHO initially designated a VOC. After which, it took 0–14 days (6.75 days ±8.4 days) for the VOC to be initially detected in Türkiye using NGS (Table 1; Figure 3).

**Table 1:** Dates of, and length of time to, detection of each VOC in Türkiye after distribution of variant-specific kits.

WHO Nomenclature	Pangolin Lineage	Detected country	Date of detection (globally)	Date of VOC designation	Date of detection in Türkiye	How long it took to detect in Türkiye via NGS	
						compared globally	After VOC kit deployment
Alpha	B.1.17	United Kingdom	September 2020	December 18, 2020	December 24, 2020	3–4 months	3 days
Beta	B.1.351	South Africa	October 2020	December 18, 2020	January 21, 2021	3–4 months	7 days before
Gamma	P.1	Brazil	November 2020	January 11, 2021	January 28, 2021	3–4 months	0 days
Delta	B.1.617.2	India	October 2020	May 11, 2021	a) March 9, 2021 b) May 7, 2021	a) 4–5 months b) 6–7 months	a) 19 days b) 78 days
Omicron	B.1.1.529	South Africa	November 2021	November 24, 2021	December 12, 2021	2–6 weeks	5 days

The benefit of using variant-specific RT-qPCR kits is highlighted best by Türkiye’s detection of the Gamma variant (Table 1). The variant-specific RT-qPCR kit was developed 17 days after WHO designated the VOC, and on the day of kit deployment, the first Gamma case was detected using NGS, which highlights the rapid turn-around-time in an emergency setting. The variant-specific RT-qPCR kit was distributed across the country on the same day, allowing Türkiye to rapidly and inexpensively detect subsequent cases of the VOC. However, the Beta VOC was identified without targeted sequencing, suggesting it was detected as it was introduced to the country (Table 1).

In Türkiye, like most countries globally, the detection of the Delta variant on March 9, 2020, occurred unknowingly much earlier than the date WHO designated it a VOC or its first submission to online databases such as GISAID. A detailed look into the GISAID repository shows that the samples collected in March (n = 333) were not submitted until September 2021. Therefore, in Table 1, two dates are indicated for the detection of the Delta variant, the first of which was only 19 days after the Delta variant-specific kit was designed to detect the L452R mutation (Figure 3; Table 1).

To elucidate the influence of the sampling strategy on the sequences detected, the sequencing results aggregated weekly, and the RT-qPCR variant data collected between April 16, 2021, and January 30, 2022, were analyzed (Supplementary Figures S1, S2). The RT-qPCR and sequencing results differed, especially during the Delta wave. The RT-qPCR results indicate a varying proportion (40–75%) of “Other” variants during the Delta wave (week 31 – week 50). However, compared with sequencing results in the same period, all sequences generated at the NVRL were detected as Delta VOC.

### SARS-CoV-2 genomic epidemiology

#### Description of SARS-CoV-2 genomic data

As new variants emerged, the MoH-TR increased its sequencing capacity to track VOCs, validate RT-qPCR kits, and contribute NGS data to global databases, ensuring that Turkish data was reflected globally. The 86,429 sequences were filtered to include only those linked with geographical information and of high quality, resulting in 43,494 and 31,629 samples, respectively, which amount to 50.3% and 36.6% of all sequences uploaded. The 31,629 samples (referred to as 31 k) are the primary focus of the following analysis.

The 31 k samples were assigned 225 different pangolin lineages (Supplementary Table S1). The Delta VOC accounted for up to 83% of the 31 k sequences, which were uploaded with high-quality geographical data (*n* = 26,336). Other than VOCs (Alpha; *n* = 410, Beta; *n* = 624, Gamma; n = 89, Omicron; *n* = 31), specific variants of interest (VOIs) were also detected in Türkiye (e.g., Epsilon *n* = 2; Eta *n* = 31; Kappa *n* = 1). However, 4,105 samples sequenced were classified as no particular VOC or VOI, termed “Other,” which amounts to >12% of all sequences uploaded.

Sample-to-result data from the 31,629 cases show that Türkiye’s median time from sample collection to uploading sequencing data to global databases was 18 days, with a mean of less than 25 days (Supplementary Figure S3).

The laboratory lacked a robust data storage infrastructure to store FASTA sequences, so it used GISAID as a data storage and reporting database. If a sequence was required for further analysis, the relevant sample sequence connected to the NVRL code(s) was downloaded from GISAID. Therefore, all Turkish SARS-CoV-2 genomic data was obtained from GISAID for this study.

There was high variability in sequences generated monthly throughout 2020–22. The monthly case numbers in Türkiye were compared to (i) the total amount of sequences generated (86 k dataset), (ii) the total amount of sequences that had geographical information, and (iii) high-quality sequences with geographical information in Table 2. Notably, during April 2021, sequencing numbers were lowest, while Türkiye experienced a significant increase in case numbers, with only 0.01% of positive cases sequenced that month. This was followed by an incremental increase starting in June, which peaked in August 2021. Over half of all sequenced samples were sequenced in August 2021 (n = 43,494, 52% of all uploaded genomes, 6.83% of all positive cases monthly) (Table 2). This was prompted by calls from the international community to increase the number of sequences uploaded to GISAID to relax travel restrictions and boost tourism. However, due to the ruptured linkage of samples to metadata, only 14% of these samples had associated geographical information and could be used for this analysis. Using the Pearson, Spearman, and Kendall correlation test, no correlation was observed between case and sequencing numbers in any of the three cohorts.

**Table 2:** Sequencing data uploaded to GISAID and the percentage of those uploaded with linked geographical information aggregated by month.

Epidemiological Month	Positive COVID-19 cases	86 k cohort (total sequences uploaded to GISAID)	86 k cohort (sequenced genomes per COVID-19 cases)	43 k cohort (%)	31 k cohort (%)
2020_12	71,594	24	0.03%	79.17%	25.00%
2021_1	299,518	557	0.19%	98.56%	63.38%
2021_2	242,065	1923	0.79%	89.39%	79.77%
2021_3	644,786	2,703	0.42%	95.49%	84.65%
2021_4*	1,579,153	189	0.01%	74.07%	72.49%
2021_5	470,615	582	0.12%	97.42%	94.67%
2021_6	191,525	2088	1.09%	91.71%	87.12%
2021_7	303,198	6,640	2.19%	61.97%	59.98%
2021_8*	661,286	45,152	6.83%	14.11%	12.70%
2021_9	765,739	6,537	0.85%	95.56%	85.02%
2021_10	878,918	2,330	0.27%	92.75%	68.80%
2021_11	762,600	5,121	0.67%	99.65%	75.41%
2021_12	686,962	6,301	0.92%	98.71%	64.75%
2022_1*	2,137,332	4,895	0.23%	97.83%	1.94%
2022_2*	1,733,794	1,367	0.08%	73.15%	1.76%
TOTAL	11,429,085	86,429	0.76%	50.32%	36.60%

Sequencing quality also varied throughout the period of this study. Samples sequenced in January and February 2022 had ~98% lower quality than previous months, as seen in Table 1, rows 2022_1 and 2022_2. To investigate the reason behind the decrease, the quality of sequences generated per geographical region and VOC was analyzed (Supplementary Figure S4). In general, no decrease in sequencing quality was observed for most VOIs and VOCs (other than Eta). However, over 95% of the Omicron sequences generated and uploaded to GISAID in the first two months of 2022 were of low quality, preventing any downstream analysis of Omicron sequences.

The 31 k samples originated from all seven geographical regions in Türkiye. A time-separated comparison of case numbers to sequencing numbers per geographic region, although limited by the availability of metadata, assisted in understanding the geographical representation of sequences (Supplementary Figure S5). The Aegean Region was the largest contributor of sequences during September 2021, although case numbers were lower than the Marmara and Central Anatolia regions. From September to December 2021, the Aegean Region was the majority contributor to the national sample sequences. Additionally, although case numbers of both the Black Sea and Central Anatolia regions were similar, sequencing numbers were much higher from the Black Sea region.

#### Spatiotemporal distribution of VOC and VOI of SARS-CoV-2

**Figure 5:**
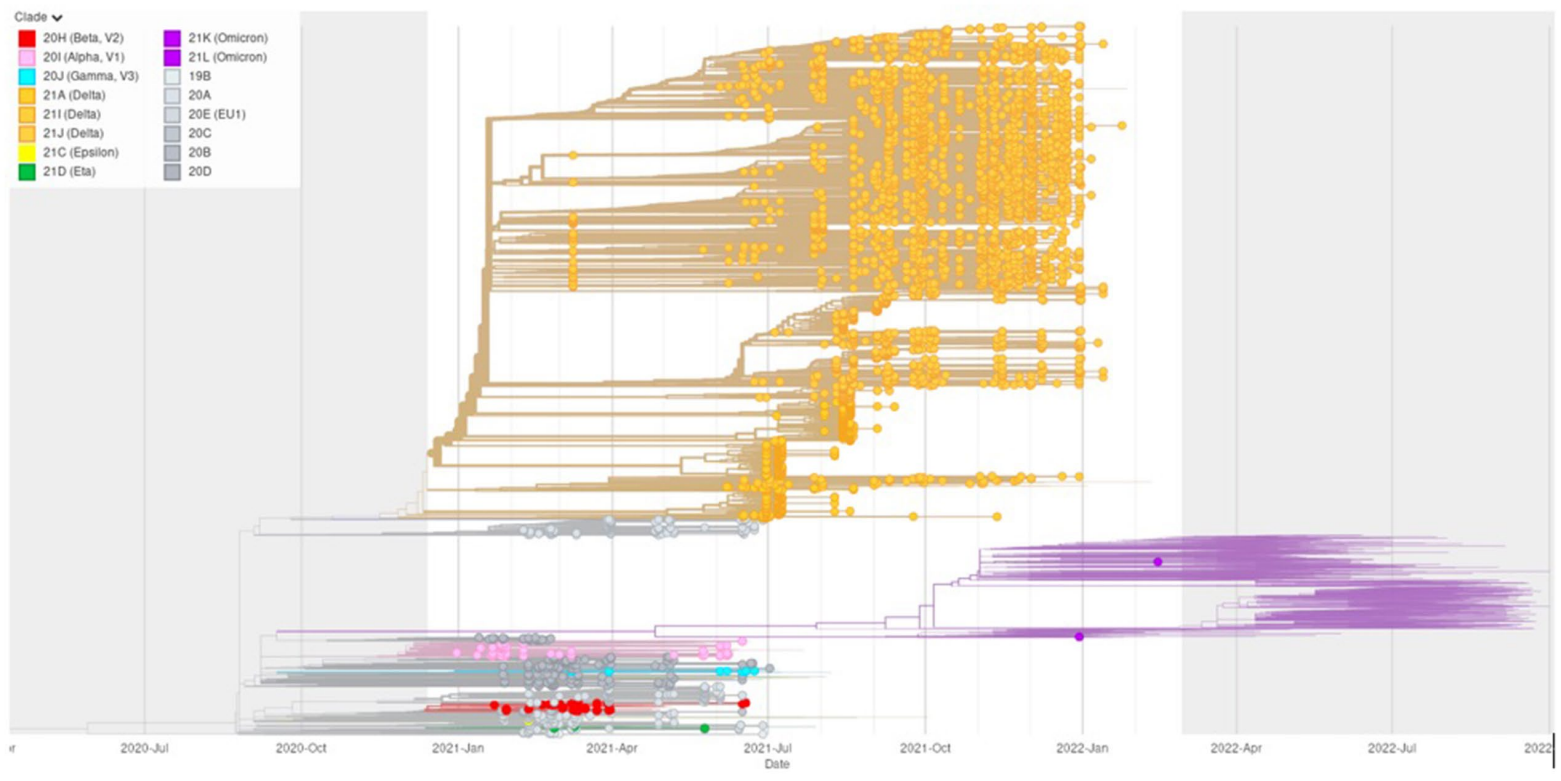
Time-resolved phylogenetic tree inferred for a randomly down-sampled (~*n* = 5,000) 31 k high-quality sequence data set against a regional non-Turkish European background of (~*n* = 1,000) and a global non-European (~*n* = 1,000) context of samples. Clade assignation by Nextstrain Clades was colored corresponding to the color code used throughout this study for the WHO nomenclature variants of concern (VOC: Alpha: pink, Beta: red, Delta: orange, Gamma: cyan, and Omicron: purple) and the variants of interest (VOI: Epsilon: yellow and Eta: green). The time frame of this study between December 28, 2020, and February 14, 2022, is highlighted by a white background compared to the grey background for context samples detected before and after the study period.

Out of the 31 k (*n* = 31,629) data set, 5,000 samples were randomly chosen by probabilistically down-sampling from their distribution over the geographical regions of Türkiye and collection months. These subsets of strains were used for phylogenetic tree construction. The phylogenetic tree inferred by Nextstrain’s augur pipeline and visualized by its auspice tool is displayed in Figure 5.

This time-resolved phylogeny shows that within the first seven months of the second year of the COVID-19 pandemic, four VOCs (VOC: Alpha, Beta, Delta, and Gamma), as well as two VOIs (VOI: Epsilon and Eta) co-circulated together with various Nextstrain clades (19B, 20A, 20E (EU1), 20C, 20B, and 20D) whose respective Pangolin lineages were not classified as VOC or VOI classes under the WHO nomenclature (grey). While Nextstrain clades belonging to the Delta VOC were detected in Türkiye as early as March 2021, they replaced this mixed group of previously co-circulating VOC and VOI classes entirely from July 2021 onwards. Thereafter, the Omicron VOC took over at the beginning of the pandemic’s third year (January 2022).

As plotted against their European and global context in Figure 5, samples classified as VOC categories Alpha, Beta, and Gamma, as well as VOI classes Epsilon and Eta, were detected several months later (in January and February 2021) than their detection in other countries. In comparison to regional and global detection of VOC Omicron, which occurred in October 2021, Türkiye’s first cases were confirmed in January and February 2022, according to available data.

#### International and national transmission patterns of VOCs and VOIs

**Figure 6:**
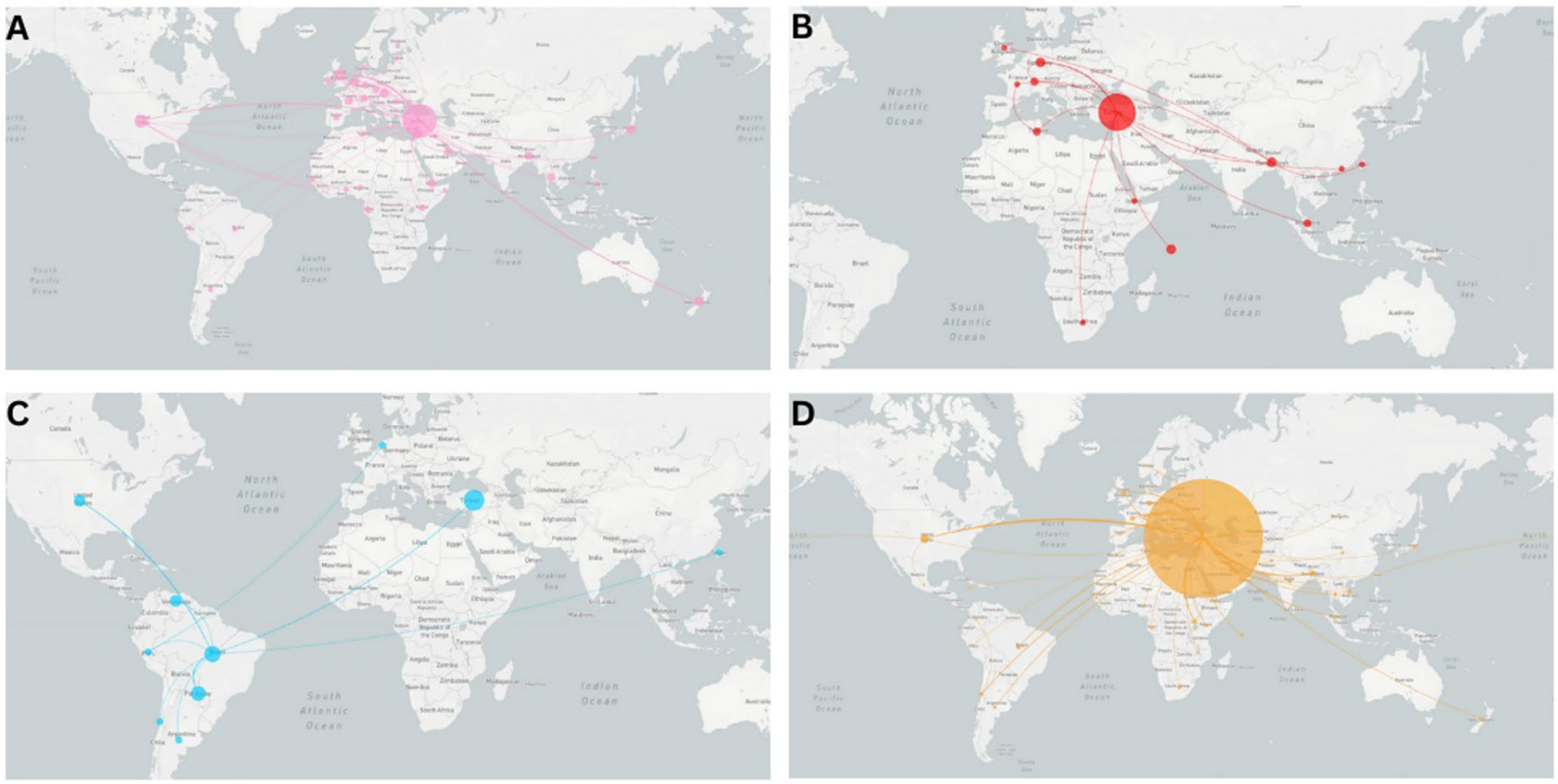
Global introduction to, and transmission from, Türkiye of VOCs. Transmission from origin to destination is indicated as a counter-clockwise curve direction. The thickness of the line symbolizes the number of exchanges. **(A)** Alpha variant, **(B)** Beta variant, **(C)** Gamma variant, and **(D)** Delta variant.

The data indicate that Türkiye’s geographical position as a travel hub played a role in transmitting each VOC globally, even with travel restrictions implemented (Figure 6). Based on the limited data included in the analysis, this is indicated by the counter-clockwise orientation of the transmission lines starting from Türkiye at the center of the map. The analysis suggests that the Alpha variant was introduced to Türkiye from two separate countries, the UK and Israel (Figure 6A), and Türkiye played a role in its subsequent global transmission. Transmission events of Alpha VOC occurred from Türkiye to the United Kingdom, the US, New Zealand, and several European, Southern American, and Asian countries (Figure 6A). While the Beta VOC originated in South Africa in October 2020, ongoing transmission to other European, African, and Asian countries occurred from Türkiye (Figure 6B). In contrast to the Alpha, Beta, and Delta VOCs, the data indicates that the Gamma VOC was introduced to Türkiye with no further international transmission (Figure 6C). The Delta VOC, as indicated by thicker transmission lines, was transmitted from Türkiye to several countries (Figure 6D). Due to the small number of Omicron sequences in the 31 k cohort, there were not enough sequences to infer geographical transmission.

**Figure 7:**
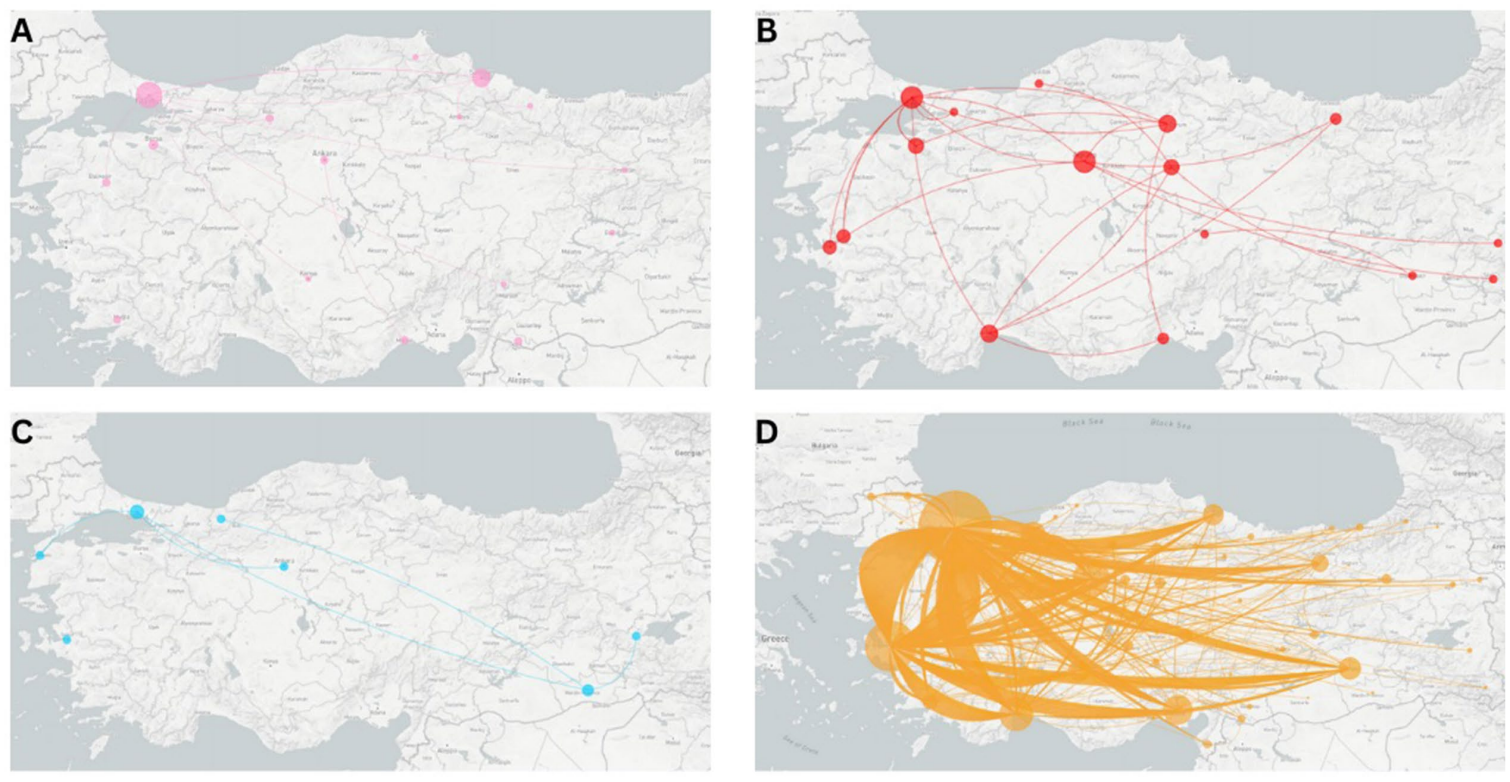
National introduction to, and further transmission of, VOCs in Turkiye. Transmission from origin to destination is indicated as a counter-clockwise curve direction. The thickness of the line symbolizes the number of exchanges. **(A)** Alpha variant, **(B)** Beta variant, **(C)** Gamma variant, and **(D)** Delta variant.

National trends of VOC transmission within Türkiye (Figure 7) suggest that most transmissions began from Istanbul to other provinces. The Alpha VOC was also transmitted from Samsun to surrounding provinces, and a thick transmission line indicates substantial transmission from Samsun to Istanbul. For the Beta variant, Istanbul, Ankara, and Antalya acted as sources of transmission around the country (Figure 7B). Further transmission was noted from Mardin further east and to the east of the Marmara region (Figure 7C). As displayed by large circles and thick counter-clockwise transmission lines in Figure 7D, the transmission patterns of Delta VOC within the country identified Istanbul, followed by Izmir, as the two main sources of transmission to other provinces of Türkiye.

## Discussion

The COVID-19 pandemic emphasized the significant role of genomic surveillance in identifying and monitoring the spread of the virus and estimating its rate of evolution. Türkiye’s approach to diagnosing and sequencing SARS-CoV-2 using a combination of variant specific RT-qPCR and NGS demonstrated adaptability and flexibility and provides insights for LMICs without established genomic surveillance systems. This study charts Türkiye’s journey in developing genomic surveillance capacity, despite minimal prior familiarity with NGS technology. Through an analysis of testing and genomic sequencing data spanning the initial two years of the pandemic, we underscore the successes and challenges encountered and below we reflect on lessons important for other countries.

### Testing strategy

Our findings highlight the importance of establishing a comprehensive testing strategy as early as possible in an epidemic/pandemic, with clear reporting lines for all laboratories. In Türkiye, close collaboration with the private sector, enabling the development of variant-specific RT-qPCR kits throughout the pandemic, was an important advantage over other settings (37–39). However, we acknowledge that this may not have been possible in countries with stricter regulations on collaboration with the private sector and outside of emergency conditions. The initial collaboration with the private sector to develop the Wuhan-1 SARS-CoV-2 RT-qPCR kit was helpful because it enabled the national production and distribution of variant kits, allowing Türkiye to rapidly establish extensive diagnostics capacity. Given the global demand early in the pandemic for commercially available kits and the fractured supply chain, this was a significant advantage in Türkiye. Following this initial success, continued collaboration allowed Türkiye to rapidly develop and implement kits to screen for novel VOCs as they were introduced and transmitted around the country. This rapid process enabled the detection and tracking of VOCs using RT-qPCR and enabled the screening of high numbers of samples for VOCs, which meant that only a small proportion of samples required NGS. Alongside sequencing, these kits permitted Türkiye to detect and characterize circulating variants and to conduct targeted and consequently cost-effective sequencing.

### Sequencing sampling strategy

While Türkiye’s unique testing strategy assisted in detecting VOCs, our findings suggest that it limited data analysis and the influence genomic surveillance had on public health action. A detailed study of the case numbers across regions showed that sample referral was efficient and sequence results were generated quickly compared with global turnaround time benchmarks (40). With a median time of 18 days and an average of 24 days, Türkiye’s sample referral strategy worked efficiently. This was reinforced by regular adaptation of the sampling strategy by the NVRL. However, this adaptive system also meant that the sequenced samples were not geographically representative, with over and under-sequencing occurring across Türkiye’s seven regions. Due to increased workload and pandemic conditions, staff at the regional laboratories had difficulty adhering to the defined sampling strategy developed by the NVRL. This resulted in high variability in monthly sequencing numbers and geographical representativeness of samples, limiting the range of sequences generated and, subsequently, the analysis that could be performed. For optimal use of sequencing data, sampling testing strategy and sample representativeness are important influences on the detection and characterization of circulating strains. The discrepancy between RT-qPCR and sequencing results reinforces the importance of a clear and concise sampling strategy for easy adherence. Combined with variant RT-qPCR assays, regular random sampling, supplemented by targeted sequencing, could have provided a more accurate picture of the circulating strains.

### Data quality

Türkiye’s experience highlighted the importance of good laboratory practice to ensure the high quality of data generated and shared globally, which must be adhered to, even in emergencies. In Türkiye’s situation, sequences generated were of adequate quality throughout most of the pandemic. However, a significant decrease in the quality of sequences uploaded to GISAID was noted for the Omicron VOC cases, investignation into the cause of which was beyond the scope of this study. However, as reported globally (41), this drop in sequence quality could be attributed to the high number of mutations that the Omicron VOC displays, resulting in decreased amplicon generation. This claim is supported by the fact that no significant decrease in the quality of Delta VOC sequences was noted within the same period. The drop in data quality highlights two important points. Firstly, genomic surveillance laboratories should have adequate bioinformatic quality control measures in place to detect any decrease in the quality of sequencing before uploading to global databases. Secondly, genomic surveillance staff should be well-informed of all novel mutations and update laboratory procedures accordingly to ensure sequencing quality.

### Metadata linkage and data storage

Establishing an appropriate data management system for novel data can be challenging during an emergency. In Türkiye, the focus on identifying VOIs and VOCs, took priority over ensuring that metadata standards were defined and upheld. The subsequent lack of metadata limited the application of genomic sequencing data for epidemiological analysis, and guiding public health action. This highlights the complexity of data linkage in a developing genomic surveillance system, even in settings with a robust health information management system. Although over 0.5% of all cases were sequenced and uploaded to global databases, in line with suggested standards (40), geographical metadata was not linked for over 50% of sequences. The NVRL had an in-house laboratory information management system, which was not integrated into the broader health information management system, preventing metadata linkage to sequenced samples, which limited the usefulness of genomic surveillance in informing real-time responses. This challenge was not unique to Türkiye and is an important lesson for other countries. Genomic sequencing data must be linked to epidemiological, clinical and other metadata through robust data management systems to have meaningful public health impact.

### Phylodynamic analysis

Türkiye’s biggest city, Istanbul, hosts one of the world’s largest airports. Therefore, it is not surprising that transmission of SARS-CoV-2 VOCs occurred from Türkiye to other countries globally, especially European countries. Nationally, as expected, major cities in Türkiye, like Istanbul, Izmir, Ankara, and Antalya, played a significant role in the transmission of the virus around the county. However, some cities, such as Samsun in the Black Sea region and Mardin in the Southeastern Anatolia region, also played a role in transmitting VOCs. Although previous studies have compared Turkish to European SARS-CoV-2 strains, they have not been of the same scale (42). Importantly, the Beta variant appears to have been transmitted globally from Türkiye. However, Türkiye detected it much later than other countries, emphasizing the importance of a robust system for tracking and detecting variants, including at points of entry. Although routine testing of passengers arriving in the country was conducted at the Istanbul airport, these samples were not routinely submitted for sequencing. Additionally, our phylogeographic analysis showed an independent introduction of the Alpha VOC near the border with Syria, highlighting the importance of border testing.

### Building a national genomic surveillance system

Since 2021, the MoH-TR and the WHO country office in Türkiye have collaborated closely to increase NGS, bioinformatics, molecular epidemiology, and data management capacities to address some of the challenges highlighted here. Importantly, Türkiye has developed a five-year national genomic surveillance strategy to integrate all areas of infectious disease genomic surveillance in Türkiye, enhancing preparedness for future health emergencies. These efforts are guided by the Global Genomic Surveillance Strategy for Pathogens with Pandemic and Epidemic Potential, 2022–2032, published by the WHO in 2022 (43). Throughout 2021–23, significant investments have been made in sequencing equipment, computing infrastructure, training of personnel, and data management and sharing. National data management and data storage infrastructure is being developed within the MoH-TR, which would link each regional laboratory to the centralized NVRL, and sequencing capacity is being increased at the regional level in Adana (Mediterranean region) and Erzurum (Eastern Anatolia region). In addition, the LIMS is now integrated into the Health Information System (HIMS), which links metadata through an automated system.

### Limitations of the study

Our study has several limitations. Firstly, the analysis of geographical representation conducted from samples obtained within the time frame of this study was conducted using only a fraction (n = 31,629), approximately 36%, of the total sequences generated (n = 86,429) in Türkiye. Without the complete 86 k dataset, we acknowledge that inferring geographical representativeness is flawed. Secondly, the exact detection date of the Delta variant is unclear. Thirdly, inadequate documentation of samples referred to the NVRL for sequencing limited assessment of how well each of the 16 laboratories adhered to the sampling strategy. Finally, no patient-related metadata, such as gender, age, vaccination, previous infection status, etc., were available, which hampered further genomic epidemiological analysis.

## Conclusion

This paper provides valuable insights into the testing and genomic surveillance systems adopted by Türkiye during the COVID-19 pandemic and offers lessons for other LMICs without prior genomic sequencing capacity. The findings underscore the need for robust testing and sampling strategies, streamlined sample referral, and integrated data management with metadata linkage and data quality crucial for impactful epidemiological analysis. We recommend developing national genomic surveillance strategies to guide sustainable and integrated expansion of capacities built for COVID-19 and to optimize the effective utilization of sequencing data for public health action.

## Data availability statement

Publicly available datasets were analyzed in this study. This data can be found here: All sequencing data was down loaded from GISAID.

## Ethics statement

Ethical approval was not required for the studies involving humans because this work was conducted as part of the public health response to the COVID-19 pandemic, led by the MoH-TR. The studies were conducted in accordance with the local legislation and institutional requirements. All SARS-CoV-2 sequences and information (e.g. collection date, location) included in this analysis were obtained as part of the first-line testing, analysis, and sequencing of samples from suspected cases through the national outbreak response. All patient samples were de-identified and no other individual-specific information was used in this study. Written informed consent to participate in this study was not required from the participants or the participants' legal guardians/next of kin in accordance with national legislation and the institutional requirements.

## Author contributions

SY: Methodology, Writing – review & editing, Data curation, Investigation. YC: Data curation, Investigation, Writing – review & editing. ED: Data curation, Writing – review & editing, Formal analysis, Methodology, Visualization, Writing – original draft. KK: Formal analysis, Methodology, Visualization, Writing – original draft, Writing – review & editing. FB: Writing – review & editing, Data curation, Investigation. GÜ: Data curation, Writing – review & editing, Formal analysis, Methodology. BM: Writing – review & editing, Conceptualization. ES: Writing – review & editing. GK: Writing – review & editing, Conceptualization, Project administration, Resources, Supervision. PR: Conceptualization, Project administration, Resources, Supervision, Writing – review & editing, Funding acquisition, Methodology, Visualization. SK: Writing – review & editing.
