# Supplementary material for: Genomic surveillance during the first two years of the COVID-19 pandemic – country experience and lessons learned from Türkiye
Source: Front Public Health. 2024 May 24;12:1332109. doi: 10.3389/fpubh.2024.1332109 (PMC11160438; doi:10.3389/fpubh.2024.1332109)
Supplement: Supplementary file 1 [file Data_Sheet_1.docx]

# Supplementary Information

## Supplementary Methods:

### The testing of Türkiye

#### 2020 - Before variant PCR kits were developed.

Prior to creating a national qPCR kit, the NVRLT was the only diagnostics center in the country for SARS-CoV-2. The NVRLT, in collaboration with a private company, developed a national PCR kit which was distributed nationwide and used as the official kit in all authorized labs. This kit required 40 cycles and a positive sample was detected at <38 Cq. For more detailed information, please refer here. Initially, all positive samples were sent to the NVRL when case numbers were manageable. However, as cases increased, only suspicious samples were referred to the NVRLT for validation by regional labs.

#### 2021 & 2022 - After variant PCR was introduced.

We created variant kits, and the first PCR variant generated matched a routine SARS-CoV-2 sequencing sample referred to the NVRL. The NVRL paused test result validation due to the need for more workers for NGS applications. The regional influenza surveillance lab typically sends samples to the NVRL for sequencing. Table 1 below shows an example of the sequencing sampling plan in Turkey when using the V3 kit, the longest-running PCR kit in the country.

| **Case Number** | **FAM (SARS-CoV-2, ORF1+N)** | **ROX (S_E484K)** | **CY5 (N_D3L)** | **CY5.5 (S_L452R)** | **Result** | **Action Taken** |
| --- | --- | --- | --- | --- | --- | --- |
| **Case 1** | **-** | **-** | **-** | **-** | **1)** SARS-CoV-2 negative | Reported as negative |
| **Case 2** | **+** | **-** | **-** | **-** | **1)** SARS-CoV-2 positive, **2)** variant negative | All samples sent to the sequencing |
| **Case 3** | **+** | **+** | **-** | **-** | **1)** SARS-CoV-2 positive, **2)** variants that contain the E484K mutation (Beta/Gamma) are positive, **3)** The Alpha variant and variants that contain L452R are negative (Delta) | 10% of samples sent to sequencing |
| **Case 4** | **+** | **+** | **+** | **-** | **1)** SARS-CoV-2 positive, **2)** Alpha variant that contains E484K mutation are positive, and **3)** variants containing L452R are negative. | 10% of samples sent to sequencing |
| **Case 5** | **+** | **+** | **-** | **+** | **1)** SARS-CoV-2 pozitive. **2)** Variants that contain both E484K and L452R is positive **3)** B.1.1.7 negative. | 10% of samples sent to sequencing |
| **Case 6** | **+** | **-** | **+** | **+** | **1)** SARS-CoV-2 positive. **2)** B.1.1.7 positive. **3)** variants containing E484K and L452R negative. | 10% of samples sent to sequencing |
| **Case 7** | **+** | **-** | **-** | **+** | **1)** SARS-CoV-2 pozitive. **2)** variants that contain L452R mutation is positive. **3)** B.1.1.7 and variants that contain E484K are negative. | 10% of samples sent to sequencing |
| **Case 8** | **+** | **-** | **+** | **+** | **1)** SARS-CoV-2 pozitive. **2)** variants that contain L452R mutation is positive. **3)** variants that contain E484K are negative. | 10% of samples sent to sequencing |

#### Algorithm for detection

- If Cq-FAM ≤ 33 - positive, else - negative
- If Cq-ROX ≤ 33 - positive, else - negative
- If Cq-Cy5 ≤ 33 - positive, else - negative
- If Cq-Cy5.5≤ 33 - Calculate [Cq_Cy5.5 – Cq_FAM] & [Cq_Cy5.5 – Cq_Cy5]. If [Cq_Cy5.5 – Cq_FAM] <4 & [Cq_Cy5.5 – Cq_Cy5] <0 - Sample is Cy5.5 positive. Else - Cy5.5 negative.

## Supplementary Figures:

### Supplementary Figure 1


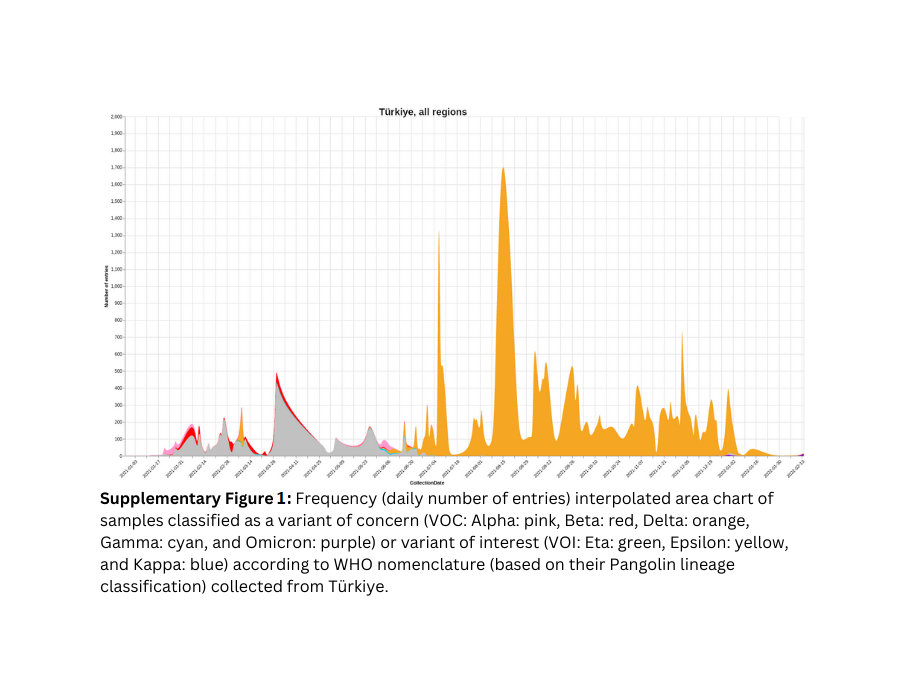


### Supplementary Figure 2


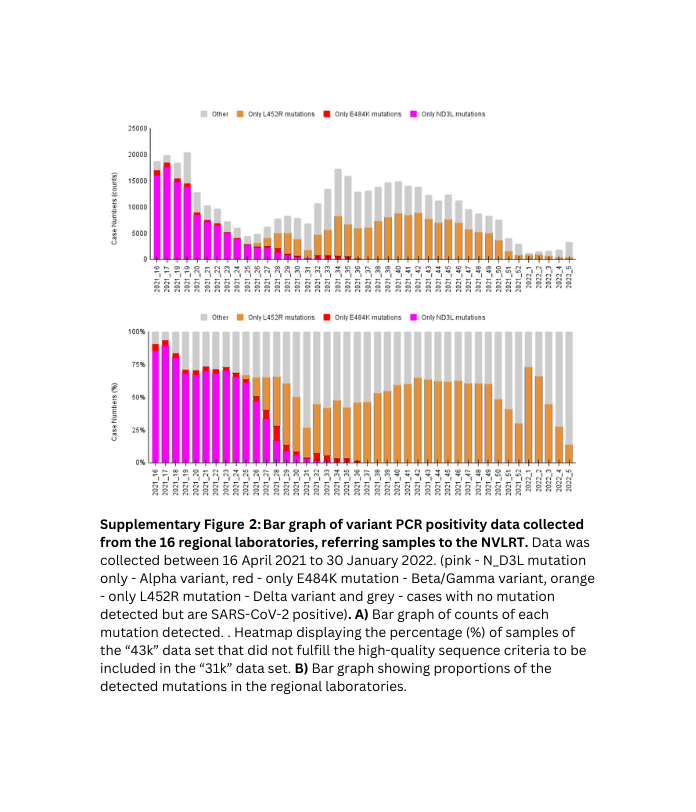


### Supplementary Figure 3


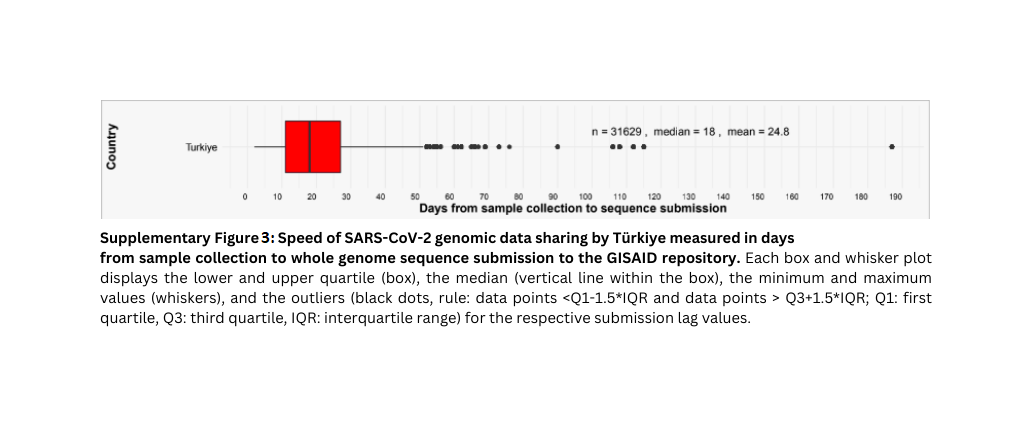


### Supplementary Figure 4


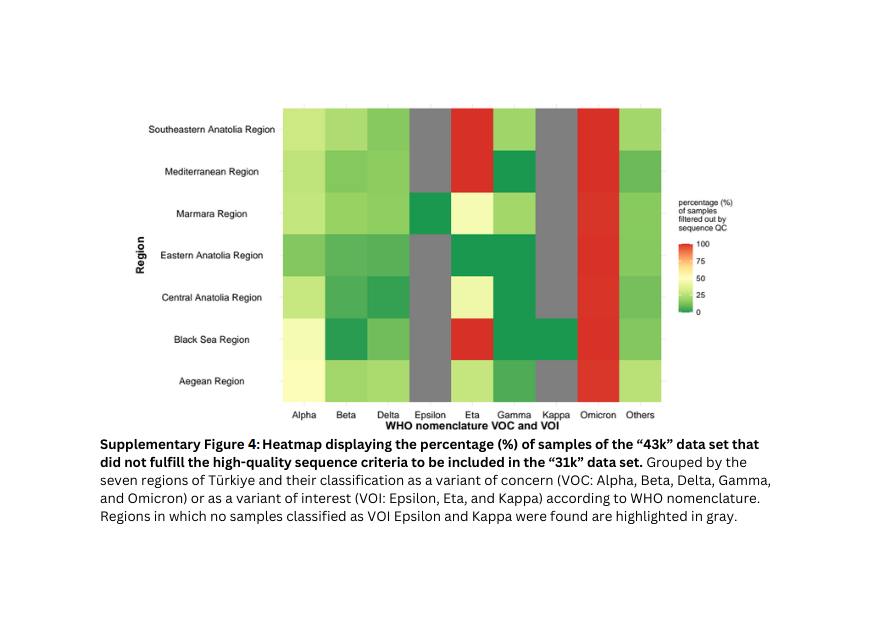


### Supplementary Figure 5


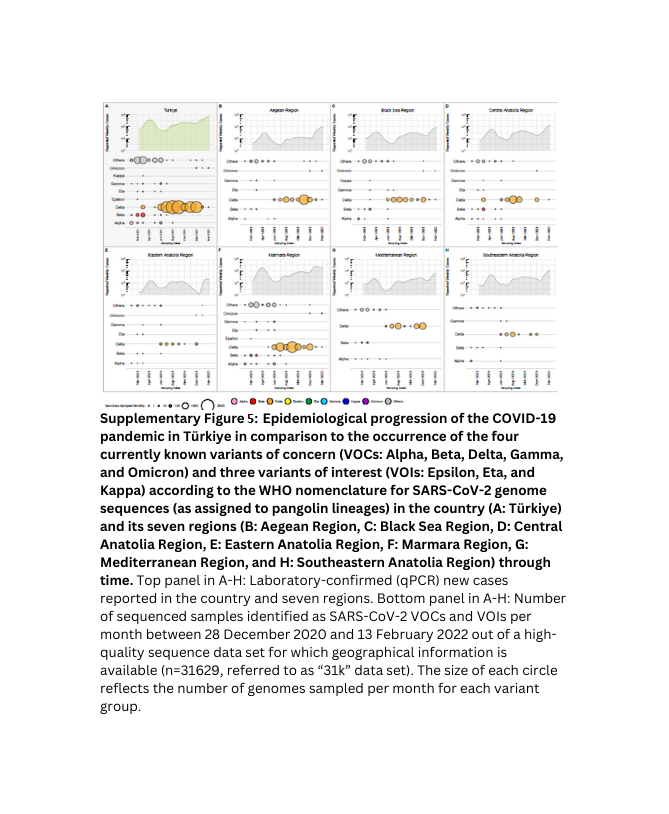


## Supplementary Tables:

### Supplementary Table 1

Description of SARS-CoV-2 lineages observed in Türkiye.

| **WHO Nomenclature** | **Pangolin Lineage** | **#of samples in 86k** | **#of samples 43k** | **#of samples 31k** | **% decrease of samples w/ geodata** | **% decrease of high quality of samples w/ geodata** |
| --- | --- | --- | --- | --- | --- | --- |
| **Alpha** | B.1.1.7 | 2177 | 615 | 410 | -71,75% | -81,17% |
|  | Q.1 | 1 | 1 | 0 | 0,00% | -100,00% |
| **Beta** | B.1.351 | 800 | 704 | 618 | -12,00% | -22,75% |
|  | B.1.351.2 | 10 | 8 | 6 | -20,00% | -40,00% |
| **Delta** | B.1.617.2 | 20156 | 8778 | 7845 | -56,45% | -61,08% |
|  | AY.121 | 6385 | 5628 | 4676 | -11,86% | -26,77% |
|  | AY.43 | 16566 | 4334 | 4092 | -73,84% | -75,30% |
|  | AY.126 | 5675 | 4458 | 3280 | -21,44% | -42,20% |
|  | AY.122 | 4442 | 2769 | 2237 | -37,66% | -49,64% |
|  | AY.112 | 1446 | 1258 | 1047 | -13,00% | -27,59% |
|  | AY.46 | 542 | 388 | 336 | -28,41% | -38,01% |
|  | AY.33 | 523 | 376 | 321 | -28,11% | -38,62% |
|  | AY.46.2 | 489 | 383 | 320 | -21,68% | -34,56% |
|  | AY.127 | 366 | 328 | 266 | -10,38% | -27,32% |
|  | AY.9.2 | 525 | 265 | 262 | -49,52% | -50,10% |
|  | AY.39 | 217 | 212 | 168 | -2,30% | -22,58% |
|  | AY.4.6 | 218 | 176 | 136 | -19,27% | -37,61% |
|  | AY.20 | 155 | 147 | 111 | -5,16% | -28,39% |
|  | AY.106 | 159 | 122 | 106 | -23,27% | -33,33% |
|  | AY.4 | 144 | 137 | 104 | -4,86% | -27,78% |
|  | AY.71 | 215 | 108 | 95 | -49,77% | -55,81% |
|  | AY.78 | 245 | 119 | 95 | -51,43% | -61,22% |
|  | AY.44 | 491 | 85 | 82 | -82,69% | -83,30% |
|  | AY.113 | 103 | 86 | 81 | -16,50% | -21,36% |
|  | AY.42 | 88 | 84 | 74 | -4,55% | -15,91% |
|  | AY.65 | 122 | 56 | 54 | -54,10% | -55,74% |
|  | AY.129 | 72 | 65 | 53 | -9,72% | -26,39% |
|  | AY.125 | 55 | 50 | 43 | -9,09% | -21,82% |
|  | AY.46.4 | 52 | 46 | 36 | -11,54% | -30,77% |
|  | AY.100 | 44 | 36 | 27 | -18,18% | -38,64% |
|  | AY.84 | 20 | 20 | 17 | 0,00% | -15,00% |
|  | AY.92 | 19 | 19 | 16 | 0,00% | -15,79% |
|  | AY.109 | 19 | 17 | 15 | -10,53% | -21,05% |
|  | AY.131 | 19 | 17 | 15 | -10,53% | -21,05% |
|  | AY.5 | 18 | 18 | 15 | 0,00% | -16,67% |
|  | AY.104 | 29 | 29 | 14 | 0,00% | -51,72% |
|  | AY.120 | 15 | 15 | 12 | 0,00% | -20,00% |
|  | AY.124 | 18 | 18 | 12 | 0,00% | -33,33% |
|  | AY.23 | 13 | 12 | 12 | -7,69% | -7,69% |
|  | AY.29.1 | 12 | 12 | 12 | 0,00% | 0,00% |
|  | AY.91.1 | 22 | 12 | 12 | -45,45% | -45,45% |
|  | AY.102 | 20 | 20 | 11 | 0,00% | -45,00% |
|  | AY.103 | 52 | 22 | 11 | -57,69% | -78,85% |
|  | AY.128 | 13 | 11 | 10 | -15,38% | -23,08% |
|  | AY.4.11 | 10 | 10 | 10 | 0,00% | 0,00% |
|  | AY.34 | 18 | 18 | 9 | 0,00% | -50,00% |
|  | AY.45 | 51 | 11 | 9 | -78,43% | -82,35% |
|  | AY.95 | 28 | 9 | 9 | -67,86% | -67,86% |
|  | AY.114 | 11 | 8 | 8 | -27,27% | -27,27% |
|  | AY.123 | 11 | 9 | 8 | -18,18% | -27,27% |
|  | AY.16 | 14 | 12 | 8 | -14,29% | -42,86% |
|  | AY.75 | 21 | 8 | 8 | -61,90% | -61,90% |
|  | AY.36 | 11 | 10 | 7 | -9,09% | -36,36% |
|  | AY.73 | 16 | 7 | 7 | -56,25% | -56,25% |
|  | AY.105 | 9 | 9 | 5 | 0,00% | -44,44% |
|  | AY.7.1 | 9 | 9 | 5 | 0,00% | -44,44% |
|  | AY.94 | 5 | 5 | 5 | 0,00% | 0,00% |
|  | AY.98 | 7 | 7 | 5 | 0,00% | -28,57% |
|  | AY.117 | 4 | 4 | 4 | 0,00% | 0,00% |
|  | AY.37 | 16 | 5 | 4 | -68,75% | -75,00% |
|  | AY.46.6 | 5 | 5 | 4 | 0,00% | -20,00% |
|  | AY.6 | 5 | 5 | 4 | 0,00% | -20,00% |
|  | AY.83 | 5 | 5 | 4 | 0,00% | -20,00% |
|  | AY.88 | 5 | 4 | 4 | -20,00% | -20,00% |
|  | AY.119 | 4 | 3 | 3 | -25,00% | -25,00% |
|  | AY.122.1 | 3 | 3 | 3 | 0,00% | 0,00% |
|  | AY.133 | 4 | 4 | 3 | 0,00% | -25,00% |
|  | AY.32 | 3 | 3 | 3 | 0,00% | 0,00% |
|  | AY.35 | 21 | 3 | 3 | -85,71% | -85,71% |
|  | AY.4.2.3 | 4 | 4 | 3 | 0,00% | -25,00% |
|  | AY.4.4 | 4 | 4 | 3 | 0,00% | -25,00% |
|  | AY.68 | 7 | 4 | 3 | -42,86% | -57,14% |
|  | AY.72 | 4 | 4 | 3 | 0,00% | -25,00% |
|  | AY.107 | 2 | 2 | 2 | 0,00% | 0,00% |
|  | AY.110 | 3 | 3 | 2 | 0,00% | -33,33% |
|  | AY.111 | 4 | 4 | 2 | 0,00% | -50,00% |
|  | AY.116 | 6 | 4 | 2 | -33,33% | -66,67% |
|  | AY.122.2 | 8 | 2 | 2 | -75,00% | -75,00% |
|  | AY.25.1 | 7 | 3 | 2 | -57,14% | -71,43% |
|  | AY.27 | 2 | 2 | 2 | 0,00% | 0,00% |
|  | AY.3 | 2 | 2 | 2 | 0,00% | 0,00% |
|  | AY.30 | 4 | 2 | 2 | -50,00% | -50,00% |
|  | AY.34.1 | 7 | 7 | 2 | 0,00% | -71,43% |
|  | AY.36.1 | 4 | 3 | 2 | -25,00% | -50,00% |
|  | AY.60 | 2 | 2 | 2 | 0,00% | 0,00% |
|  | AY.70 | 4 | 4 | 2 | 0,00% | -50,00% |
|  | AY.79 | 2 | 2 | 2 | 0,00% | 0,00% |
|  | AY.81 | 2 | 2 | 2 | 0,00% | 0,00% |
|  | AY.98.1 | 3 | 3 | 2 | 0,00% | -33,33% |
|  | AY.1 | 9 | 8 | 1 | -11,11% | -88,89% |
|  | AY.119.2 | 1 | 1 | 1 | 0,00% | 0,00% |
|  | AY.17 | 1 | 1 | 1 | 0,00% | 0,00% |
|  | AY.29 | 1 | 1 | 1 | 0,00% | 0,00% |
|  | AY.3.1 | 1 | 1 | 1 | 0,00% | 0,00% |
|  | AY.39.1.4 | 2 | 2 | 1 | 0,00% | -50,00% |
|  | AY.4.7 | 9 | 2 | 1 | -77,78% | -88,89% |
|  | AY.48 | 1 | 1 | 1 | 0,00% | 0,00% |
|  | AY.50 | 3 | 1 | 1 | -66,67% | -66,67% |
|  | AY.51 | 7 | 1 | 1 | -85,71% | -85,71% |
|  | AY.54 | 2 | 1 | 1 | -50,00% | -50,00% |
|  | AY.66 | 2 | 2 | 1 | 0,00% | -50,00% |
|  | AY.7 | 2 | 2 | 1 | 0,00% | -50,00% |
|  | AY.75.2 | 5 | 1 | 1 | -80,00% | -80,00% |
|  | AY.77 | 1 | 1 | 1 | 0,00% | 0,00% |
|  | AY.80 | 1 | 1 | 1 | 0,00% | 0,00% |
|  | AY.86 | 2 | 1 | 1 | -50,00% | -50,00% |
|  | AY.9 | 1 | 1 | 1 | 0,00% | 0,00% |
|  | AY.91 | 1 | 1 | 1 | 0,00% | 0,00% |
|  | AY.118 | 3 | 3 | 0 | 0,00% | -100,00% |
|  | AY.34.2 | 1 | 1 | 0 | 0,00% | -100,00% |
|  | AY.39.1 | 2 | 2 | 0 | 0,00% | -100,00% |
|  | AY.4.2 | 1 | 1 | 0 | 0,00% | -100,00% |
|  | AY.4.2.1 | 4 | 4 | 0 | 0,00% | -100,00% |
|  | AY.47 | 1 | 1 | 0 | 0,00% | -100,00% |
|  | AY.61 | 1 | 0 | 0 | -100,00% | -100,00% |
|  | AY.82 | 2 | 2 | 0 | 0,00% | -100,00% |
|  | AY.85 | 1 | 1 | 0 | 0,00% | -100,00% |
|  | AY.99 | 3 | 1 | 0 | -66,67% | -100,00% |
| **Epsilon** | B.1.427 | 2 | 2 | 2 | 0,00% | 0,00% |
| **Eta** | B.1.525 | 91 | 60 | 31 | -34,07% | -65,93% |
| **Gamma** | P.1 | 156 | 79 | 65 | -49,36% | -58,33% |
|  | P.1.16 | 20 | 19 | 19 | -5,00% | -5,00% |
|  | P.1.1 | 3 | 2 | 2 | -33,33% | -33,33% |
|  | P.1.17 | 6 | 2 | 2 | -66,67% | -66,67% |
|  | P.1.14 | 1 | 1 | 1 | 0,00% | 0,00% |
|  | P.1.15 | 1 | 1 | 0 | 0,00% | -100,00% |
| **Kappa** | B.1.617.1 | 9 | 1 | 1 | -88,89% | -88,89% |
| **Mu** | B.1.621 | 2 | 0 | 0 | -100,00% | -100,00% |
| **NonVOCassigned** | B.1 | 12711 | 2594 | 2363 | -79,59% | -81,41% |
|  | B.1.1 | 2104 | 1313 | 882 | -37,60% | -58,08% |
|  | B.1.1.189 | 180 | 165 | 155 | -8,33% | -13,89% |
|  | B.1.619 | 169 | 90 | 83 | -46,75% | -50,89% |
|  | B.1.469 | 68 | 65 | 61 | -4,41% | -10,29% |
|  | B.1.1.317 | 65 | 40 | 40 | -38,46% | -38,46% |
|  | B.1.177 | 36 | 33 | 32 | -8,33% | -11,11% |
|  | B.1.36.1 | 40 | 38 | 31 | -5,00% | -22,50% |
|  | B.1.36 | 35 | 31 | 29 | -11,43% | -17,14% |
|  | B | 40 | 30 | 26 | -25,00% | -35,00% |
|  | B.1.1.521 | 27 | 22 | 22 | -18,52% | -18,52% |
|  | B.1.1.1 | 24 | 24 | 21 | 0,00% | -12,50% |
|  | B.1.218 | 25 | 23 | 21 | -8,00% | -16,00% |
|  | A.28 | 23 | 21 | 20 | -8,70% | -13,04% |
|  | B.1.438 | 34 | 19 | 18 | -44,12% | -47,06% |
|  | B.1.1.409 | 21 | 19 | 17 | -9,52% | -19,05% |
|  | B.1.1.119 | 258 | 15 | 14 | -94,19% | -94,57% |
|  | B.1.1.318 | 50 | 39 | 13 | -22,00% | -74,00% |
|  | B.1.160 | 16 | 14 | 13 | -12,50% | -18,75% |
|  | B.1.221 | 15 | 12 | 12 | -20,00% | -20,00% |
|  | B.1.36.10 | 12 | 12 | 11 | 0,00% | -8,33% |
|  | B.1.1.136 | 9 | 9 | 9 | 0,00% | 0,00% |
|  | B.1.523 | 9 | 9 | 9 | 0,00% | 0,00% |
|  | B.1.1.525 | 10 | 9 | 8 | -10,00% | -20,00% |
|  | B.1.470 | 9 | 9 | 8 | 0,00% | -11,11% |
|  | C.36.3 | 18 | 9 | 8 | -50,00% | -55,56% |
|  | B.1.1.294 | 7 | 7 | 7 | 0,00% | 0,00% |
|  | B.1.36.8 | 10 | 7 | 7 | -30,00% | -30,00% |
|  | B.1.1.282 | 11 | 11 | 6 | 0,00% | -45,45% |
|  | R.1 | 13 | 6 | 6 | -53,85% | -53,85% |
|  | B.1.1.174 | 6 | 5 | 5 | -16,67% | -16,67% |
|  | B.1.1.274 | 5 | 5 | 5 | 0,00% | 0,00% |
|  | B.1.1.397 | 5 | 5 | 5 | 0,00% | 0,00% |
|  | B.1.1.413 | 12 | 5 | 5 | -58,33% | -58,33% |
|  | B.1.177.86 | 7 | 6 | 5 | -14,29% | -28,57% |
|  | B.1.36.7 | 6 | 5 | 5 | -16,67% | -16,67% |
|  | C.36 | 12 | 5 | 5 | -58,33% | -58,33% |
|  | B.1.1.205 | 21 | 6 | 4 | -71,43% | -80,95% |
|  | B.1.1.236 | 5 | 5 | 4 | 0,00% | -20,00% |
|  | B.1.1.243 | 6 | 5 | 4 | -16,67% | -33,33% |
|  | B.1.160.16 | 5 | 4 | 4 | -20,00% | -20,00% |
|  | B.1.258 | 4 | 4 | 4 | 0,00% | 0,00% |
|  | B.1.480 | 4 | 4 | 4 | 0,00% | 0,00% |
|  | A.27 | 11 | 7 | 3 | -36,36% | -72,73% |
|  | B.1.1.232 | 5 | 5 | 3 | 0,00% | -40,00% |
|  | B.1.1.325 | 3 | 3 | 3 | 0,00% | 0,00% |
|  | B.1.1.419 | 11 | 4 | 3 | -63,64% | -72,73% |
|  | B.1.177.10 | 3 | 3 | 3 | 0,00% | 0,00% |
|  | B.1.240 | 5 | 3 | 3 | -40,00% | -40,00% |
|  | B.1.9.5 | 4 | 3 | 3 | -25,00% | -25,00% |
|  | A.29 | 13 | 2 | 2 | -84,62% | -84,62% |
|  | AS.2 | 3 | 3 | 2 | 0,00% | -33,33% |
|  | B.1.1.10 | 2 | 2 | 2 | 0,00% | 0,00% |
|  | B.1.1.237 | 2 | 2 | 2 | 0,00% | 0,00% |
|  | B.1.1.288 | 6 | 2 | 2 | -66,67% | -66,67% |
|  | B.1.1.307 | 2 | 2 | 2 | 0,00% | 0,00% |
|  | B.1.1.37 | 2 | 2 | 2 | 0,00% | 0,00% |
|  | B.1.1.372 | 2 | 2 | 2 | 0,00% | 0,00% |
|  | B.1.1.523 | 3 | 3 | 2 | 0,00% | -33,33% |
|  | B.1.177.47 | 2 | 2 | 2 | 0,00% | 0,00% |
|  | B.1.177.73 | 2 | 2 | 2 | 0,00% | 0,00% |
|  | B.1.401 | 2 | 2 | 2 | 0,00% | 0,00% |
|  | B.1.637 | 5 | 2 | 2 | -60,00% | -60,00% |
|  | B.1.9 | 2 | 2 | 2 | 0,00% | 0,00% |
|  | C.38 | 10 | 3 | 2 | -70,00% | -80,00% |
|  | C.4 | 2 | 2 | 2 | 0,00% | 0,00% |
|  | B.1.1.137 | 1 | 1 | 1 | 0,00% | 0,00% |
|  | B.1.1.141 | 1 | 1 | 1 | 0,00% | 0,00% |
|  | B.1.1.144 | 1 | 1 | 1 | 0,00% | 0,00% |
|  | B.1.1.148 | 2 | 2 | 1 | 0,00% | -50,00% |
|  | B.1.1.161 | 1 | 1 | 1 | 0,00% | 0,00% |
|  | B.1.1.192 | 1 | 1 | 1 | 0,00% | 0,00% |
|  | B.1.1.198 | 1 | 1 | 1 | 0,00% | 0,00% |
|  | B.1.1.218 | 2 | 1 | 1 | -50,00% | -50,00% |
|  | B.1.1.220 | 1 | 1 | 1 | 0,00% | 0,00% |
|  | B.1.1.277 | 1 | 1 | 1 | 0,00% | 0,00% |
|  | B.1.1.28 | 7 | 2 | 1 | -71,43% | -85,71% |
|  | B.1.1.296 | 1 | 1 | 1 | 0,00% | 0,00% |
|  | B.1.1.312 | 1 | 1 | 1 | 0,00% | 0,00% |
|  | B.1.1.378 | 1 | 1 | 1 | 0,00% | 0,00% |
|  | B.1.1.398 | 1 | 1 | 1 | 0,00% | 0,00% |
|  | B.1.1.464 | 1 | 1 | 1 | 0,00% | 0,00% |
|  | B.1.1.507 | 1 | 1 | 1 | 0,00% | 0,00% |
|  | B.1.1.74 | 2 | 2 | 1 | 0,00% | -50,00% |
|  | B.1.1.89 | 4 | 2 | 1 | -50,00% | -75,00% |
|  | B.1.1.99 | 1 | 1 | 1 | 0,00% | 0,00% |
|  | B.1.110 | 1 | 1 | 1 | 0,00% | 0,00% |
|  | B.1.177.4 | 2 | 2 | 1 | 0,00% | -50,00% |
|  | B.1.177.77 | 1 | 1 | 1 | 0,00% | 0,00% |
|  | B.1.2 | 1 | 1 | 1 | 0,00% | 0,00% |
|  | B.1.227 | 1 | 1 | 1 | 0,00% | 0,00% |
|  | B.1.23 | 5 | 1 | 1 | -80,00% | -80,00% |
|  | B.1.235 | 1 | 1 | 1 | 0,00% | 0,00% |
|  | B.1.264.1 | 1 | 1 | 1 | 0,00% | 0,00% |
|  | B.1.284 | 1 | 1 | 1 | 0,00% | 0,00% |
|  | B.1.349 | 1 | 1 | 1 | 0,00% | 0,00% |
|  | B.1.395 | 20 | 1 | 1 | -95,00% | -95,00% |
|  | B.1.438.2 | 1 | 1 | 1 | 0,00% | 0,00% |
|  | B.1.450 | 1 | 1 | 1 | 0,00% | 0,00% |
|  | B.1.468 | 1 | 1 | 1 | 0,00% | 0,00% |
|  | B.1.511 | 1 | 1 | 1 | 0,00% | 0,00% |
|  | B.1.545 | 1 | 1 | 1 | 0,00% | 0,00% |
|  | B.1.575 | 1 | 1 | 1 | 0,00% | 0,00% |
|  | B.1.596 | 1 | 1 | 1 | 0,00% | 0,00% |
|  | B.1.630 | 4 | 2 | 1 | -50,00% | -75,00% |
|  | C.16 | 1 | 1 | 1 | 0,00% | 0,00% |
|  | C.23 | 1 | 1 | 1 | 0,00% | 0,00% |
|  | A | 1 | 1 | 0 | 0,00% | -100,00% |
|  | B.1.1.171 | 1 | 1 | 0 | 0,00% | -100,00% |
|  | B.1.1.254 | 1 | 0 | 0 | -100,00% | -100,00% |
|  | B.1.1.306 | 1 | 1 | 0 | 0,00% | -100,00% |
|  | B.1.1.442 | 4 | 4 | 0 | 0,00% | -100,00% |
|  | B.1.160.15 | 1 | 1 | 0 | 0,00% | -100,00% |
|  | B.1.177.62 | 1 | 1 | 0 | 0,00% | -100,00% |
|  | B.1.177.83 | 2 | 0 | 0 | -100,00% | -100,00% |
|  | B.1.236 | 1 | 0 | 0 | -100,00% | -100,00% |
|  | B.1.497 | 1 | 1 | 0 | 0,00% | -100,00% |
|  | B.1.640.1 | 1 | 1 | 0 | 0,00% | -100,00% |
|  | Unassigned | 8 | 5 | 0 | -37,50% | -100,00% |
| **Omicron** | B.1.1.529 | 221 | 86 | 11 | -61,09% | -95,02% |
|  | BA.1 | 5251 | 5026 | 10 | -4,28% | -99,81% |
|  | BA.2 | 230 | 197 | 10 | -14,35% | -95,65% |
|  | BA.1.1 | 579 | 522 | 0 | -9,84% | -100,00% |
|  | BA.1.1.1 | 47 | 43 | 0 | -8,51% | -100,00% |
|  | BA.1.1.14 | 2 | 1 | 0 | -50,00% | -100,00% |
|  | BA.1.1.15 | 2 | 2 | 0 | 0,00% | -100,00% |
|  | BA.1.1.16 | 1 | 1 | 0 | 0,00% | -100,00% |
|  | BA.1.1.18 | 1 | 1 | 0 | 0,00% | -100,00% |
|  | BA.1.1.7 | 2 | 0 | 0 | -100,00% | -100,00% |
|  | BA.1.10 | 2 | 1 | 0 | -50,00% | -100,00% |
|  | BA.1.13 | 1 | 1 | 0 | 0,00% | -100,00% |
|  | BA.1.14 | 14 | 14 | 0 | 0,00% | -100,00% |
|  | BA.1.14.2 | 2 | 2 | 0 | 0,00% | -100,00% |
|  | BA.1.15 | 34 | 34 | 0 | 0,00% | -100,00% |
|  | BA.1.15.1 | 10 | 10 | 0 | 0,00% | -100,00% |
|  | BA.1.16 | 1 | 1 | 0 | 0,00% | -100,00% |
|  | BA.1.17 | 71 | 69 | 0 | -2,82% | -100,00% |
|  | BA.1.17.2 | 32 | 31 | 0 | -3,13% | -100,00% |
|  | BA.1.18 | 18 | 15 | 0 | -16,67% | -100,00% |
|  | BA.1.19 | 1 | 1 | 0 | 0,00% | -100,00% |
|  | BA.1.20 | 2 | 2 | 0 | 0,00% | -100,00% |
|  | BA.1.5 | 1 | 1 | 0 | 0,00% | -100,00% |
|  | BA.1.9 | 18 | 15 | 0 | -16,67% | -100,00% |
|  | BA.2.3 | 7 | 7 | 0 | 0,00% | -100,00% |
|  | BA.2.5 | 4 | 1 | 0 | -75,00% | -100,00% |
|  | BA.2.9 | 12 | 9 | 0 | -25,00% | -100,00% |
| **Total** | | **86429** | **43494** | **31629** | **-49,68%** | **-63,40%** |
