# Supplementary material for: Genomic surveillance during the first two years of the COVID-19 pandemic – country experience and lessons learned from Türkiye
Source: Front Public Health. 2024 May 24;12:1332109. doi: 10.3389/fpubh.2024.1332109 (PMC11160438; doi:10.3389/fpubh.2024.1332109)
Supplement: Supplementary file 2 [file Data_Sheet_2.docx]

## **Supplementary Information**

### Supplementary Figure 1


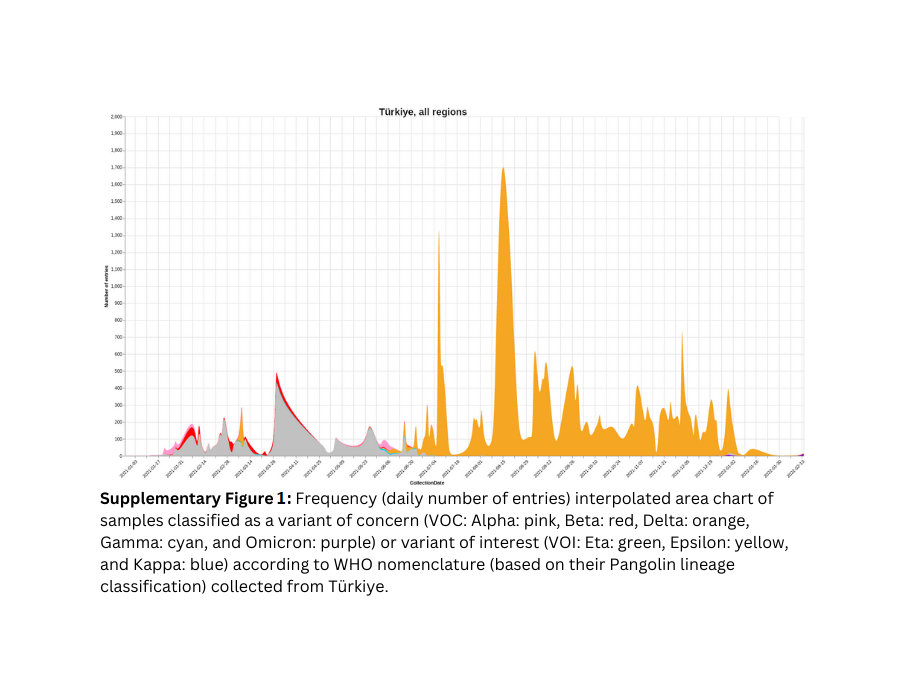


### Supplementary Figure 2


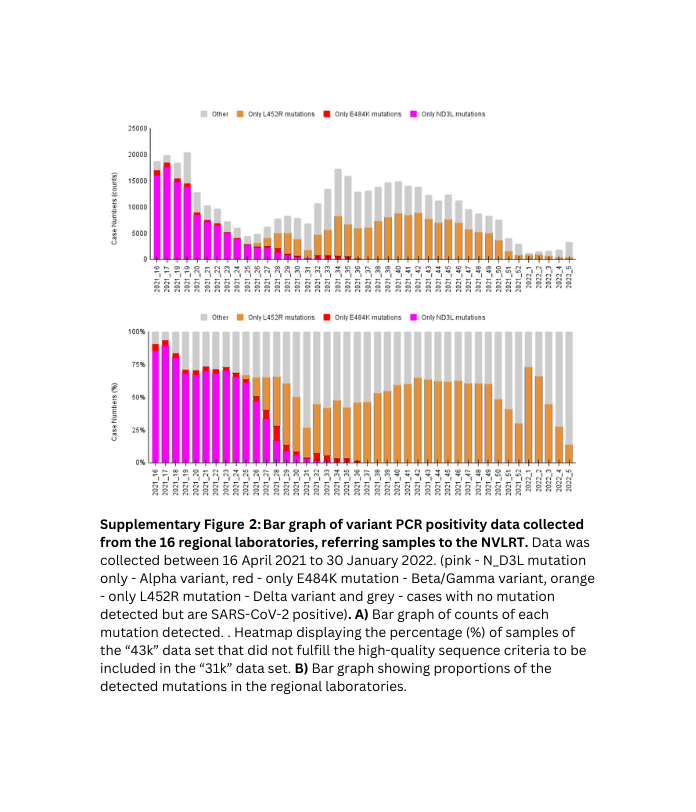


### Supplementary Figure 3


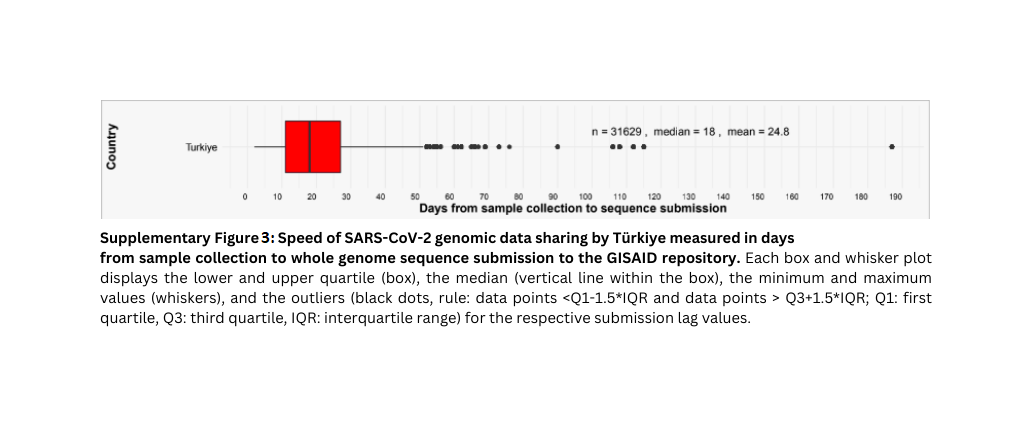


### Supplementary Figure 4


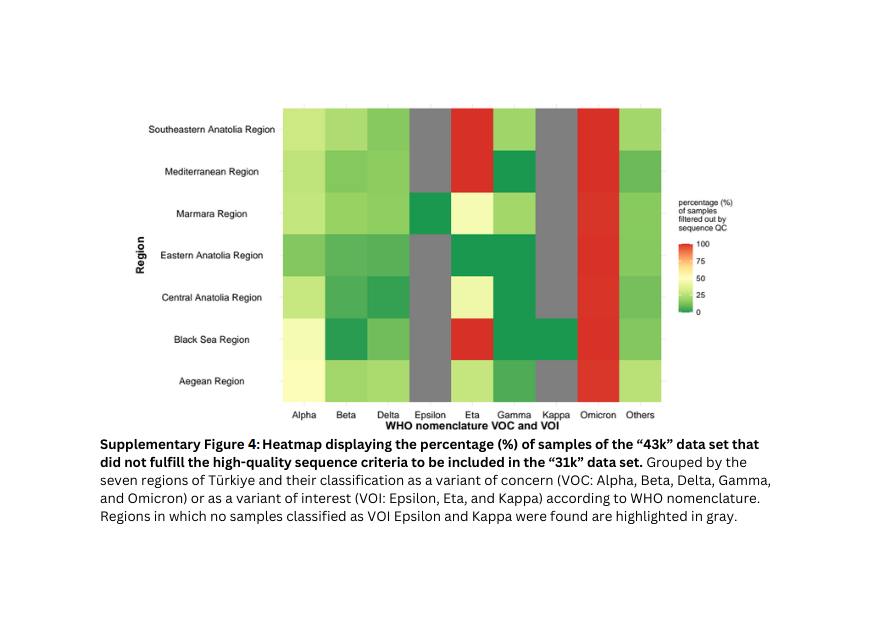


### Supplementary Figure 5


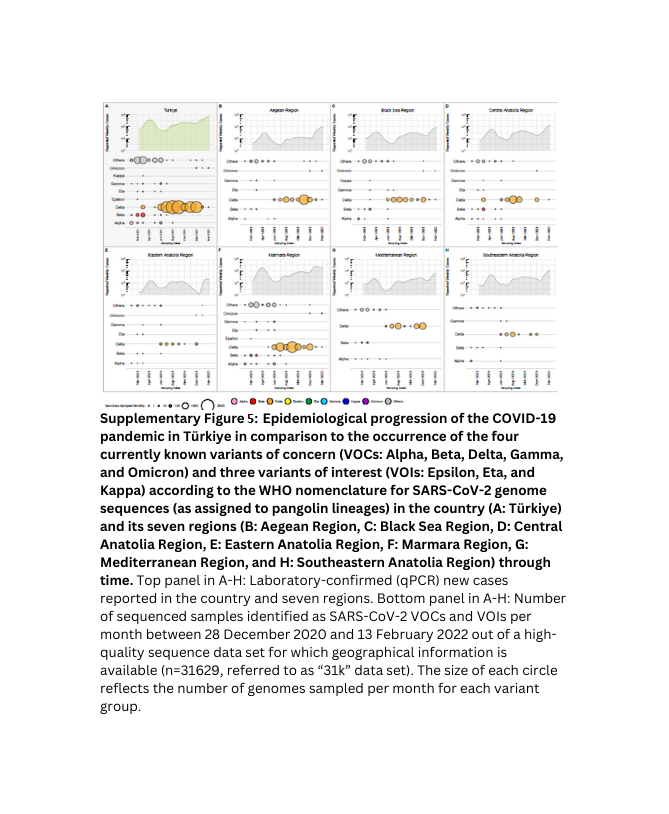


### Supplementary Table 1

Description of SARS-CoV-2 lineages detected in Türkiye.

| **WHO Nomenclature** | **Pangolin Lineage** | **#of samples in 86k** | **#of samples 43k** | **#of samples 31k** | **% decrease of samples w/ geodata** | **% decrease of high quality of samples w/ geodata** |
| --- | --- | --- | --- | --- | --- | --- |
| **Alpha** | B.1.1.7 | 2177 | 615 | 410 | -71,75% | -81,17% |
|  | Q.1 | 1 | 1 | 0 | 0,00% | -100,00% |
| **Beta** | B.1.351 | 800 | 704 | 618 | -12,00% | -22,75% |
|  | B.1.351.2 | 10 | 8 | 6 | -20,00% | -40,00% |
| **Delta** | B.1.617.2 | 20156 | 8778 | 7845 | -56,45% | -61,08% |
|  | AY.121 | 6385 | 5628 | 4676 | -11,86% | -26,77% |
|  | AY.43 | 16566 | 4334 | 4092 | -73,84% | -75,30% |
|  | AY.126 | 5675 | 4458 | 3280 | -21,44% | -42,20% |
|  | AY.122 | 4442 | 2769 | 2237 | -37,66% | -49,64% |
|  | AY.112 | 1446 | 1258 | 1047 | -13,00% | -27,59% |
|  | AY.46 | 542 | 388 | 336 | -28,41% | -38,01% |
|  | AY.33 | 523 | 376 | 321 | -28,11% | -38,62% |
|  | AY.46.2 | 489 | 383 | 320 | -21,68% | -34,56% |
|  | AY.127 | 366 | 328 | 266 | -10,38% | -27,32% |
|  | AY.9.2 | 525 | 265 | 262 | -49,52% | -50,10% |
|  | AY.39 | 217 | 212 | 168 | -2,30% | -22,58% |
|  | AY.4.6 | 218 | 176 | 136 | -19,27% | -37,61% |
|  | AY.20 | 155 | 147 | 111 | -5,16% | -28,39% |
|  | AY.106 | 159 | 122 | 106 | -23,27% | -33,33% |
|  | AY.4 | 144 | 137 | 104 | -4,86% | -27,78% |
|  | AY.71 | 215 | 108 | 95 | -49,77% | -55,81% |
|  | AY.78 | 245 | 119 | 95 | -51,43% | -61,22% |
|  | AY.44 | 491 | 85 | 82 | -82,69% | -83,30% |
|  | AY.113 | 103 | 86 | 81 | -16,50% | -21,36% |
|  | AY.42 | 88 | 84 | 74 | -4,55% | -15,91% |
|  | AY.65 | 122 | 56 | 54 | -54,10% | -55,74% |
|  | AY.129 | 72 | 65 | 53 | -9,72% | -26,39% |
|  | AY.125 | 55 | 50 | 43 | -9,09% | -21,82% |
|  | AY.46.4 | 52 | 46 | 36 | -11,54% | -30,77% |
|  | AY.100 | 44 | 36 | 27 | -18,18% | -38,64% |
|  | AY.84 | 20 | 20 | 17 | 0,00% | -15,00% |
|  | AY.92 | 19 | 19 | 16 | 0,00% | -15,79% |
|  | AY.109 | 19 | 17 | 15 | -10,53% | -21,05% |
|  | AY.131 | 19 | 17 | 15 | -10,53% | -21,05% |
|  | AY.5 | 18 | 18 | 15 | 0,00% | -16,67% |
|  | AY.104 | 29 | 29 | 14 | 0,00% | -51,72% |
|  | AY.120 | 15 | 15 | 12 | 0,00% | -20,00% |
|  | AY.124 | 18 | 18 | 12 | 0,00% | -33,33% |
|  | AY.23 | 13 | 12 | 12 | -7,69% | -7,69% |
|  | AY.29.1 | 12 | 12 | 12 | 0,00% | 0,00% |
|  | AY.91.1 | 22 | 12 | 12 | -45,45% | -45,45% |
|  | AY.102 | 20 | 20 | 11 | 0,00% | -45,00% |
|  | AY.103 | 52 | 22 | 11 | -57,69% | -78,85% |
|  | AY.128 | 13 | 11 | 10 | -15,38% | -23,08% |
|  | AY.4.11 | 10 | 10 | 10 | 0,00% | 0,00% |
|  | AY.34 | 18 | 18 | 9 | 0,00% | -50,00% |
|  | AY.45 | 51 | 11 | 9 | -78,43% | -82,35% |
|  | AY.95 | 28 | 9 | 9 | -67,86% | -67,86% |
|  | AY.114 | 11 | 8 | 8 | -27,27% | -27,27% |
|  | AY.123 | 11 | 9 | 8 | -18,18% | -27,27% |
|  | AY.16 | 14 | 12 | 8 | -14,29% | -42,86% |
|  | AY.75 | 21 | 8 | 8 | -61,90% | -61,90% |
|  | AY.36 | 11 | 10 | 7 | -9,09% | -36,36% |
|  | AY.73 | 16 | 7 | 7 | -56,25% | -56,25% |
|  | AY.105 | 9 | 9 | 5 | 0,00% | -44,44% |
|  | AY.7.1 | 9 | 9 | 5 | 0,00% | -44,44% |
|  | AY.94 | 5 | 5 | 5 | 0,00% | 0,00% |
|  | AY.98 | 7 | 7 | 5 | 0,00% | -28,57% |
|  | AY.117 | 4 | 4 | 4 | 0,00% | 0,00% |
|  | AY.37 | 16 | 5 | 4 | -68,75% | -75,00% |
|  | AY.46.6 | 5 | 5 | 4 | 0,00% | -20,00% |
|  | AY.6 | 5 | 5 | 4 | 0,00% | -20,00% |
|  | AY.83 | 5 | 5 | 4 | 0,00% | -20,00% |
|  | AY.88 | 5 | 4 | 4 | -20,00% | -20,00% |
|  | AY.119 | 4 | 3 | 3 | -25,00% | -25,00% |
|  | AY.122.1 | 3 | 3 | 3 | 0,00% | 0,00% |
|  | AY.133 | 4 | 4 | 3 | 0,00% | -25,00% |
|  | AY.32 | 3 | 3 | 3 | 0,00% | 0,00% |
|  | AY.35 | 21 | 3 | 3 | -85,71% | -85,71% |
|  | AY.4.2.3 | 4 | 4 | 3 | 0,00% | -25,00% |
|  | AY.4.4 | 4 | 4 | 3 | 0,00% | -25,00% |
|  | AY.68 | 7 | 4 | 3 | -42,86% | -57,14% |
|  | AY.72 | 4 | 4 | 3 | 0,00% | -25,00% |
|  | AY.107 | 2 | 2 | 2 | 0,00% | 0,00% |
|  | AY.110 | 3 | 3 | 2 | 0,00% | -33,33% |
|  | AY.111 | 4 | 4 | 2 | 0,00% | -50,00% |
|  | AY.116 | 6 | 4 | 2 | -33,33% | -66,67% |
|  | AY.122.2 | 8 | 2 | 2 | -75,00% | -75,00% |
|  | AY.25.1 | 7 | 3 | 2 | -57,14% | -71,43% |
|  | AY.27 | 2 | 2 | 2 | 0,00% | 0,00% |
|  | AY.3 | 2 | 2 | 2 | 0,00% | 0,00% |
|  | AY.30 | 4 | 2 | 2 | -50,00% | -50,00% |
|  | AY.34.1 | 7 | 7 | 2 | 0,00% | -71,43% |
|  | AY.36.1 | 4 | 3 | 2 | -25,00% | -50,00% |
|  | AY.60 | 2 | 2 | 2 | 0,00% | 0,00% |
|  | AY.70 | 4 | 4 | 2 | 0,00% | -50,00% |
|  | AY.79 | 2 | 2 | 2 | 0,00% | 0,00% |
|  | AY.81 | 2 | 2 | 2 | 0,00% | 0,00% |
|  | AY.98.1 | 3 | 3 | 2 | 0,00% | -33,33% |
|  | AY.1 | 9 | 8 | 1 | -11,11% | -88,89% |
|  | AY.119.2 | 1 | 1 | 1 | 0,00% | 0,00% |
|  | AY.17 | 1 | 1 | 1 | 0,00% | 0,00% |
|  | AY.29 | 1 | 1 | 1 | 0,00% | 0,00% |
|  | AY.3.1 | 1 | 1 | 1 | 0,00% | 0,00% |
|  | AY.39.1.4 | 2 | 2 | 1 | 0,00% | -50,00% |
|  | AY.4.7 | 9 | 2 | 1 | -77,78% | -88,89% |
|  | AY.48 | 1 | 1 | 1 | 0,00% | 0,00% |
|  | AY.50 | 3 | 1 | 1 | -66,67% | -66,67% |
|  | AY.51 | 7 | 1 | 1 | -85,71% | -85,71% |
|  | AY.54 | 2 | 1 | 1 | -50,00% | -50,00% |
|  | AY.66 | 2 | 2 | 1 | 0,00% | -50,00% |
|  | AY.7 | 2 | 2 | 1 | 0,00% | -50,00% |
|  | AY.75.2 | 5 | 1 | 1 | -80,00% | -80,00% |
|  | AY.77 | 1 | 1 | 1 | 0,00% | 0,00% |
|  | AY.80 | 1 | 1 | 1 | 0,00% | 0,00% |
|  | AY.86 | 2 | 1 | 1 | -50,00% | -50,00% |
|  | AY.9 | 1 | 1 | 1 | 0,00% | 0,00% |
|  | AY.91 | 1 | 1 | 1 | 0,00% | 0,00% |
|  | AY.118 | 3 | 3 | 0 | 0,00% | -100,00% |
|  | AY.34.2 | 1 | 1 | 0 | 0,00% | -100,00% |
|  | AY.39.1 | 2 | 2 | 0 | 0,00% | -100,00% |
|  | AY.4.2 | 1 | 1 | 0 | 0,00% | -100,00% |
|  | AY.4.2.1 | 4 | 4 | 0 | 0,00% | -100,00% |
|  | AY.47 | 1 | 1 | 0 | 0,00% | -100,00% |
|  | AY.61 | 1 | 0 | 0 | -100,00% | -100,00% |
|  | AY.82 | 2 | 2 | 0 | 0,00% | -100,00% |
|  | AY.85 | 1 | 1 | 0 | 0,00% | -100,00% |
|  | AY.99 | 3 | 1 | 0 | -66,67% | -100,00% |
| **Epsilon** | B.1.427 | 2 | 2 | 2 | 0,00% | 0,00% |
| **Eta** | B.1.525 | 91 | 60 | 31 | -34,07% | -65,93% |
| **Gamma** | P.1 | 156 | 79 | 65 | -49,36% | -58,33% |
|  | P.1.16 | 20 | 19 | 19 | -5,00% | -5,00% |
|  | P.1.1 | 3 | 2 | 2 | -33,33% | -33,33% |
|  | P.1.17 | 6 | 2 | 2 | -66,67% | -66,67% |
|  | P.1.14 | 1 | 1 | 1 | 0,00% | 0,00% |
|  | P.1.15 | 1 | 1 | 0 | 0,00% | -100,00% |
| **Kappa** | B.1.617.1 | 9 | 1 | 1 | -88,89% | -88,89% |
| **Mu** | B.1.621 | 2 | 0 | 0 | -100,00% | -100,00% |
| **NonVOCassigned** | B.1 | 12711 | 2594 | 2363 | -79,59% | -81,41% |
|  | B.1.1 | 2104 | 1313 | 882 | -37,60% | -58,08% |
|  | B.1.1.189 | 180 | 165 | 155 | -8,33% | -13,89% |
|  | B.1.619 | 169 | 90 | 83 | -46,75% | -50,89% |
|  | B.1.469 | 68 | 65 | 61 | -4,41% | -10,29% |
|  | B.1.1.317 | 65 | 40 | 40 | -38,46% | -38,46% |
|  | B.1.177 | 36 | 33 | 32 | -8,33% | -11,11% |
|  | B.1.36.1 | 40 | 38 | 31 | -5,00% | -22,50% |
|  | B.1.36 | 35 | 31 | 29 | -11,43% | -17,14% |
|  | B | 40 | 30 | 26 | -25,00% | -35,00% |
|  | B.1.1.521 | 27 | 22 | 22 | -18,52% | -18,52% |
|  | B.1.1.1 | 24 | 24 | 21 | 0,00% | -12,50% |
|  | B.1.218 | 25 | 23 | 21 | -8,00% | -16,00% |
|  | A.28 | 23 | 21 | 20 | -8,70% | -13,04% |
|  | B.1.438 | 34 | 19 | 18 | -44,12% | -47,06% |
|  | B.1.1.409 | 21 | 19 | 17 | -9,52% | -19,05% |
|  | B.1.1.119 | 258 | 15 | 14 | -94,19% | -94,57% |
|  | B.1.1.318 | 50 | 39 | 13 | -22,00% | -74,00% |
|  | B.1.160 | 16 | 14 | 13 | -12,50% | -18,75% |
|  | B.1.221 | 15 | 12 | 12 | -20,00% | -20,00% |
|  | B.1.36.10 | 12 | 12 | 11 | 0,00% | -8,33% |
|  | B.1.1.136 | 9 | 9 | 9 | 0,00% | 0,00% |
|  | B.1.523 | 9 | 9 | 9 | 0,00% | 0,00% |
|  | B.1.1.525 | 10 | 9 | 8 | -10,00% | -20,00% |
|  | B.1.470 | 9 | 9 | 8 | 0,00% | -11,11% |
|  | C.36.3 | 18 | 9 | 8 | -50,00% | -55,56% |
|  | B.1.1.294 | 7 | 7 | 7 | 0,00% | 0,00% |
|  | B.1.36.8 | 10 | 7 | 7 | -30,00% | -30,00% |
|  | B.1.1.282 | 11 | 11 | 6 | 0,00% | -45,45% |
|  | R.1 | 13 | 6 | 6 | -53,85% | -53,85% |
|  | B.1.1.174 | 6 | 5 | 5 | -16,67% | -16,67% |
|  | B.1.1.274 | 5 | 5 | 5 | 0,00% | 0,00% |
|  | B.1.1.397 | 5 | 5 | 5 | 0,00% | 0,00% |
|  | B.1.1.413 | 12 | 5 | 5 | -58,33% | -58,33% |
|  | B.1.177.86 | 7 | 6 | 5 | -14,29% | -28,57% |
|  | B.1.36.7 | 6 | 5 | 5 | -16,67% | -16,67% |
|  | C.36 | 12 | 5 | 5 | -58,33% | -58,33% |
|  | B.1.1.205 | 21 | 6 | 4 | -71,43% | -80,95% |
|  | B.1.1.236 | 5 | 5 | 4 | 0,00% | -20,00% |
|  | B.1.1.243 | 6 | 5 | 4 | -16,67% | -33,33% |
|  | B.1.160.16 | 5 | 4 | 4 | -20,00% | -20,00% |
|  | B.1.258 | 4 | 4 | 4 | 0,00% | 0,00% |
|  | B.1.480 | 4 | 4 | 4 | 0,00% | 0,00% |
|  | A.27 | 11 | 7 | 3 | -36,36% | -72,73% |
|  | B.1.1.232 | 5 | 5 | 3 | 0,00% | -40,00% |
|  | B.1.1.325 | 3 | 3 | 3 | 0,00% | 0,00% |
|  | B.1.1.419 | 11 | 4 | 3 | -63,64% | -72,73% |
|  | B.1.177.10 | 3 | 3 | 3 | 0,00% | 0,00% |
|  | B.1.240 | 5 | 3 | 3 | -40,00% | -40,00% |
|  | B.1.9.5 | 4 | 3 | 3 | -25,00% | -25,00% |
|  | A.29 | 13 | 2 | 2 | -84,62% | -84,62% |
|  | AS.2 | 3 | 3 | 2 | 0,00% | -33,33% |
|  | B.1.1.10 | 2 | 2 | 2 | 0,00% | 0,00% |
|  | B.1.1.237 | 2 | 2 | 2 | 0,00% | 0,00% |
|  | B.1.1.288 | 6 | 2 | 2 | -66,67% | -66,67% |
|  | B.1.1.307 | 2 | 2 | 2 | 0,00% | 0,00% |
|  | B.1.1.37 | 2 | 2 | 2 | 0,00% | 0,00% |
|  | B.1.1.372 | 2 | 2 | 2 | 0,00% | 0,00% |
|  | B.1.1.523 | 3 | 3 | 2 | 0,00% | -33,33% |
|  | B.1.177.47 | 2 | 2 | 2 | 0,00% | 0,00% |
|  | B.1.177.73 | 2 | 2 | 2 | 0,00% | 0,00% |
|  | B.1.401 | 2 | 2 | 2 | 0,00% | 0,00% |
|  | B.1.637 | 5 | 2 | 2 | -60,00% | -60,00% |
|  | B.1.9 | 2 | 2 | 2 | 0,00% | 0,00% |
|  | C.38 | 10 | 3 | 2 | -70,00% | -80,00% |
|  | C.4 | 2 | 2 | 2 | 0,00% | 0,00% |
|  | B.1.1.137 | 1 | 1 | 1 | 0,00% | 0,00% |
|  | B.1.1.141 | 1 | 1 | 1 | 0,00% | 0,00% |
|  | B.1.1.144 | 1 | 1 | 1 | 0,00% | 0,00% |
|  | B.1.1.148 | 2 | 2 | 1 | 0,00% | -50,00% |
|  | B.1.1.161 | 1 | 1 | 1 | 0,00% | 0,00% |
|  | B.1.1.192 | 1 | 1 | 1 | 0,00% | 0,00% |
|  | B.1.1.198 | 1 | 1 | 1 | 0,00% | 0,00% |
|  | B.1.1.218 | 2 | 1 | 1 | -50,00% | -50,00% |
|  | B.1.1.220 | 1 | 1 | 1 | 0,00% | 0,00% |
|  | B.1.1.277 | 1 | 1 | 1 | 0,00% | 0,00% |
|  | B.1.1.28 | 7 | 2 | 1 | -71,43% | -85,71% |
|  | B.1.1.296 | 1 | 1 | 1 | 0,00% | 0,00% |
|  | B.1.1.312 | 1 | 1 | 1 | 0,00% | 0,00% |
|  | B.1.1.378 | 1 | 1 | 1 | 0,00% | 0,00% |
|  | B.1.1.398 | 1 | 1 | 1 | 0,00% | 0,00% |
|  | B.1.1.464 | 1 | 1 | 1 | 0,00% | 0,00% |
|  | B.1.1.507 | 1 | 1 | 1 | 0,00% | 0,00% |
|  | B.1.1.74 | 2 | 2 | 1 | 0,00% | -50,00% |
|  | B.1.1.89 | 4 | 2 | 1 | -50,00% | -75,00% |
|  | B.1.1.99 | 1 | 1 | 1 | 0,00% | 0,00% |
|  | B.1.110 | 1 | 1 | 1 | 0,00% | 0,00% |
|  | B.1.177.4 | 2 | 2 | 1 | 0,00% | -50,00% |
|  | B.1.177.77 | 1 | 1 | 1 | 0,00% | 0,00% |
|  | B.1.2 | 1 | 1 | 1 | 0,00% | 0,00% |
|  | B.1.227 | 1 | 1 | 1 | 0,00% | 0,00% |
|  | B.1.23 | 5 | 1 | 1 | -80,00% | -80,00% |
|  | B.1.235 | 1 | 1 | 1 | 0,00% | 0,00% |
|  | B.1.264.1 | 1 | 1 | 1 | 0,00% | 0,00% |
|  | B.1.284 | 1 | 1 | 1 | 0,00% | 0,00% |
|  | B.1.349 | 1 | 1 | 1 | 0,00% | 0,00% |
|  | B.1.395 | 20 | 1 | 1 | -95,00% | -95,00% |
|  | B.1.438.2 | 1 | 1 | 1 | 0,00% | 0,00% |
|  | B.1.450 | 1 | 1 | 1 | 0,00% | 0,00% |
|  | B.1.468 | 1 | 1 | 1 | 0,00% | 0,00% |
|  | B.1.511 | 1 | 1 | 1 | 0,00% | 0,00% |
|  | B.1.545 | 1 | 1 | 1 | 0,00% | 0,00% |
|  | B.1.575 | 1 | 1 | 1 | 0,00% | 0,00% |
|  | B.1.596 | 1 | 1 | 1 | 0,00% | 0,00% |
|  | B.1.630 | 4 | 2 | 1 | -50,00% | -75,00% |
|  | C.16 | 1 | 1 | 1 | 0,00% | 0,00% |
|  | C.23 | 1 | 1 | 1 | 0,00% | 0,00% |
|  | A | 1 | 1 | 0 | 0,00% | -100,00% |
|  | B.1.1.171 | 1 | 1 | 0 | 0,00% | -100,00% |
|  | B.1.1.254 | 1 | 0 | 0 | -100,00% | -100,00% |
|  | B.1.1.306 | 1 | 1 | 0 | 0,00% | -100,00% |
|  | B.1.1.442 | 4 | 4 | 0 | 0,00% | -100,00% |
|  | B.1.160.15 | 1 | 1 | 0 | 0,00% | -100,00% |
|  | B.1.177.62 | 1 | 1 | 0 | 0,00% | -100,00% |
|  | B.1.177.83 | 2 | 0 | 0 | -100,00% | -100,00% |
|  | B.1.236 | 1 | 0 | 0 | -100,00% | -100,00% |
|  | B.1.497 | 1 | 1 | 0 | 0,00% | -100,00% |
|  | B.1.640.1 | 1 | 1 | 0 | 0,00% | -100,00% |
|  | Unassigned | 8 | 5 | 0 | -37,50% | -100,00% |
| **Omicron** | B.1.1.529 | 221 | 86 | 11 | -61,09% | -95,02% |
|  | BA.1 | 5251 | 5026 | 10 | -4,28% | -99,81% |
|  | BA.2 | 230 | 197 | 10 | -14,35% | -95,65% |
|  | BA.1.1 | 579 | 522 | 0 | -9,84% | -100,00% |
|  | BA.1.1.1 | 47 | 43 | 0 | -8,51% | -100,00% |
|  | BA.1.1.14 | 2 | 1 | 0 | -50,00% | -100,00% |
|  | BA.1.1.15 | 2 | 2 | 0 | 0,00% | -100,00% |
|  | BA.1.1.16 | 1 | 1 | 0 | 0,00% | -100,00% |
|  | BA.1.1.18 | 1 | 1 | 0 | 0,00% | -100,00% |
|  | BA.1.1.7 | 2 | 0 | 0 | -100,00% | -100,00% |
|  | BA.1.10 | 2 | 1 | 0 | -50,00% | -100,00% |
|  | BA.1.13 | 1 | 1 | 0 | 0,00% | -100,00% |
|  | BA.1.14 | 14 | 14 | 0 | 0,00% | -100,00% |
|  | BA.1.14.2 | 2 | 2 | 0 | 0,00% | -100,00% |
|  | BA.1.15 | 34 | 34 | 0 | 0,00% | -100,00% |
|  | BA.1.15.1 | 10 | 10 | 0 | 0,00% | -100,00% |
|  | BA.1.16 | 1 | 1 | 0 | 0,00% | -100,00% |
|  | BA.1.17 | 71 | 69 | 0 | -2,82% | -100,00% |
|  | BA.1.17.2 | 32 | 31 | 0 | -3,13% | -100,00% |
|  | BA.1.18 | 18 | 15 | 0 | -16,67% | -100,00% |
|  | BA.1.19 | 1 | 1 | 0 | 0,00% | -100,00% |
|  | BA.1.20 | 2 | 2 | 0 | 0,00% | -100,00% |
|  | BA.1.5 | 1 | 1 | 0 | 0,00% | -100,00% |
|  | BA.1.9 | 18 | 15 | 0 | -16,67% | -100,00% |
|  | BA.2.3 | 7 | 7 | 0 | 0,00% | -100,00% |
|  | BA.2.5 | 4 | 1 | 0 | -75,00% | -100,00% |
|  | BA.2.9 | 12 | 9 | 0 | -25,00% | -100,00% |
| **Total** | | **86429** | **43494** | **31629** | **-49,68%** | **-63,40%** |
